# Proteomic Signatures of SARS-CoV-2 Susceptibility in Mexican Free-tailed Bats and Their Application to Viral Surveillance

**DOI:** 10.1093/icb/icaf148

**Published:** 2025-08-15

**Authors:** Daniel J Becker, Amanda Vicente-Santos, Ariadna E Morales, Kristin E Dyer, Beckett L Olbrys, Lauren R Lock, Michael S Smotherman, Sonja C Vernes, Michael Hiller, Amanda M Adams, Brett S Phinney, Winifred F Frick, Jeffrey S Hall

**Affiliations:** School of Biological Sciences, University of Oklahoma, Norman, OK 73019, USA; School of Biological Sciences, University of Oklahoma, Norman, OK 73019, USA; LOEWE Centre for Translational Biodiversity Genomics, Frankfurt 60325, Germany; Senckenberg Research Institute, Frankfurt 60325, Germany; Faculty of Biosciences, Goethe University Frankfurt, Frankfurt 60438, Germany; ISEM, University of Montpellier, CNRS, IRD, Montpellier 34095, France; School of Biological Sciences, University of Oklahoma, Norman, OK 73019, USA; School of Biological Sciences, University of Oklahoma, Norman, OK 73019, USA; School of Biological Sciences, University of Oklahoma, Norman, OK 73019, USA; Department of Biology, Texas A&M University, College Station, TX 77843, USA; School of Biology, University of St Andrews, St Andrews KY16 9ST, UK; LOEWE Centre for Translational Biodiversity Genomics, Frankfurt 60325, Germany; Senckenberg Research Institute, Frankfurt 60325, Germany; Faculty of Biosciences, Goethe University Frankfurt, Frankfurt 60438, Germany; Bat Conservation International, Austin, TX 78746, USA; Proteomics Core Facility, UC Davis Genome Center, University of California, Davis, CA 95616, USA; Bat Conservation International, Austin, TX 78746, USA; Ecology and Evolutionary Biology, University of California, Santa Cruz, CA 95064, USA; US Geological Survey National Wildlife Health Center, Madison, WI 53711, USA

## Abstract

The increasing emergence of virulent pathogens necessitates novel approaches to predict and manage infectious disease risks. The importance of integrating observational and experimental approaches to studying host–pathogen interactions has long been recognized, as captive studies can mechanistically test hypotheses derived from field studies and identify causal factors shaping host susceptibility or tolerance of infection. However, captive experiments can also determine biomarkers of infection outcomes that could improve later interpretation of field data and identify at-risk hosts in wild populations. Such work could be especially useful in preempting or managing risks of pathogen spillover or spillback. SARS-CoV-2 emerged in humans in late 2019 and was rapidly followed by spillback into naïve wildlife, leading to both mortality events and novel enzootic cycles. Of special concern is whether SARS-CoV-2 could establish in bats in the Americas, given that sarbecoviruses coevolved with rhinolophid bats in the Eastern Hemisphere, and as coronavirus infection may exacerbate effects of white-nose syndrome. Here, we leverage residual plasma samples from a previous SARS-CoV-2 challenge study of Mexican free-tailed bats (*Tadarida brasiliensis*) to identify candidate protein biomarkers of susceptibility and test whether these can predict coronavirus risks in wild bats. We generated plasma proteomes from captive (*n* = 20; four resistant, five susceptible, 11 unchallenged) and wild (*n* = 15) bats using the S-Trap method and LC-MS/MS, identifying 475 proteins using data-independent acquisition and a species-specific genome annotation generated by the Bat1K Project. Receiver operator characteristic curves identified 27 potential biomarkers of SARS-CoV-2 susceptibility (AUC ≥ 0.8), and subsequent enrichment analyses of these proteins suggested downregulation of blood clotting and upregulation of complement activation and humoral immunity in susceptible bats. We then mined plasma proteomes from wild bats (sampled in 2022 from Bracken Cave Preserve, the largest known Mexican free-tailed bat population) to show that all candidate biomarkers were present in this population, with coefficients of variation ranging from 16 to 150% per protein. We detected coronaviruses in 20% of wild bats, with two cases of potential SARS-CoV-2 spillback. We demonstrate that at least four of these candidate susceptibility biomarkers classified bats with and without coronavirus infection in the wild. Our results inform the possible immune strategies underlying SARS-CoV-2 susceptibility in bats and give a preliminary example of how captive challenge studies can be coupled with field studies to inform zoonotic and conservation risks.

## Introduction

The increasing emergence of virulent pathogens, with notable examples including but not limited to Nipah virus, SARS coronaviruses (CoVs), and avian influenza viruses (Harvey et al. 2023; Meadows et al. 2023), has necessitated novel approaches to predict and manage infectious disease risks to human, domestic animal, and wildlife health. The importance of integrating observational and experimental approaches to studying host–pathogen interactions has long been recognized (Plowright et al. 2008; Johnson et al. 2015), with recent work emphasizing the need to couple field studies with captive challenge studies to understand the factors shaping susceptibility, tolerance of infection, and pathogen shedding (i.e., competence) (Becker and Banerjee 2023). The relationship between these epistemologies is often seen as unidirectional, with field studies generating hypotheses that can be tested with *in vitro* or *in vivo* experiments (Gonzalez et al. 2024). For example, comparative genomics using field samples found that selection for immune genes is more common in bats than other mammals and that *ISG15*, an antiviral gene contributing to hyperinflammation during SARS-CoV-2 infection in humans, shows key residue changes in rhinolophid and hipposiderid bats specifically. Transfection experiments of primate cells with bat-derived ISG15 constructs and subsequent viral challenge verified that ISG15 from these bats, but not of other bat families, suppressed SARS-CoV-2 (Morales et al. 2025). However, experimental approaches can also provide insights that ultimately improve interpretation of field data (Pepin et al. 2017). For example, a captive study of house sparrows (*Passer domesticus*) exposed to West Nile virus found that higher baseline expression of IFN-γ, a pro-inflammatory cytokine, predicted shorter infectious periods, while higher baseline expression of IL-10, an anti-inflammatory cytokine, was linked with improved tolerance (Burgan et al. 2018). Such validated biomarkers of competence could identify especially susceptible or infectious hosts in the wild, thereby facilitating prevention or management of disease risks. However, this experimental-to-field pipeline remains rare.

Such coupling of experimental and field studies is especially relevant for studying the outcomes of infection with novel pathogens, such as pathogen spillover or spillback. Spillback is broadly defined as pathogen transmission from humans to wildlife (Epstein and Price 2009), with select examples such as respiratory viruses transmitted to chimpanzees (Negrey et al. 2019) and enteric bacteria transmitted to seabirds (Cerdà-Cuéllar et al. 2019). More recently, and primarily through the context of SARS-CoV-2, spillback has referred primarily to cases in which spillover from wildlife establishes ongoing transmission cycles in humans, followed by transmission into naïve wildlife communities (Franklin and Bevins 2020). The introduction of these pathogens back into wildlife can have important implications for conservation practice and zoonotic risk, as spillback can lead to pronounced mortality events and to establishment of new enzootic cycles of infection that then serve as additional sources of human infection (Fagre et al. 2022). In the case of SARS-CoV-2, emergence in December 2019 and subsequent pandemic spread was rapidly followed by spillback into dogs (Sit et al. 2020), cats (Zhang et al. 2020), large felids in zoos (McAloose et al. 2020), and a wide range of wild mammal species (Goldberg et al. 2024). Spillback into mink (*Neovison vison*) and white-tailed deer (*Odocoileus virginianus*) has been followed by onward transmission within these new reservoir hosts (Oreshkova et al. 2020; Kuchipudi et al. 2022), with at least several potential cases of transmission back to humans (Hammer et al. 2021; Feng et al. 2023). Within mink specifically, SARS-CoV-2 also caused substantial mortality events (Eckstrand et al. 2021), highlighting the diversity of infection outcomes following spillback. However, the individual- and population-level impacts of SARS-CoV-2 infection remain poorly understood for most mammals, especially bats.

SARS-CoV-2 is particularly relevant to bats, given that this order of flying mammals is the likely ancestral source of both genera of CoVs pathogenic in humans (i.e., α- and β-CoVs) (Woo et al. 2012; Maestri et al. 2024). SARS-like CoVs (subgenus *Sarbecovirus*, within the β-CoV genus) are genetically diverse and naturally circulate in rhinolophid bats (Lau et al. 2005; Ge et al. 2013; Hu et al. 2017). Rhinolophids harbor multiple sarbecoviruses (e.g., RaTG13) with high genetic similarity to SARS-CoV-2 (e.g., 96.2%) (Zhou et al. 2020), but these viruses likely diverged from the human virus 40–70 years ago (Boni et al. 2020). As rhinolophid bats are restricted to the Palearctic, Afrotropical, and Indomalayan realms, bats without coevolutionary history with sarbecoviruses could be both more susceptible and less tolerant to SARS-CoV-2 spillback (Olival et al. 2020). Whether spillback could allow SARS-CoV-2 to establish in Nearctic, Neotropical, or Australasian bats and create new reservoirs of human infection remains largely unknown, as does the impact the virus may have on wild bats (Goldberg et al. 2024).

The latter unknown could be especially important for North American bats in the context of *Pseudogymnoascus destructans* (*Pd*), the causative agent of white-nose syndrome (WNS), which has caused dramatic population declines and even species extirpations throughout much of the United States and Canada (Frick et al. 2015; Cheng et al. 2021; Hooton et al. 2023). Infection with *Pd* and its physiological effects on bats could increase susceptibility to or impacts of SARS-CoV-2, and this novel viral infection could also exacerbate the severity of *Pd* pathology through co-infection dynamics and immune trade-offs (Davy et al. 2018; Salazar et al. 2022). While evidence of spillback into North American bats remains limited (Moran et al. 2023; Goldberg et al. 2024), the United States and Canada suffer from substantial surveillance gaps for CoVs more generally (Hernández-Aguilar et al. 2021; Cohen et al. 2023). Given the potential for synzootic interactions between SARS-CoV-2 and *Pd* (Sweeny et al. 2021), captive challenge studies have assessed susceptibility to this virus in several North American bat species. Big brown bats (*Eptesicus fuscus*) and little brown bats (*Myotis lucifugus*) are fully resistant to SARS-CoV-2 (Hall et al. 2021; 2024), whereas Mexican free-tailed bats (*Tadarida brasiliensis*) are instead susceptible, with population-level variation in the degree to which they shed virus (Bosco-Lauth et al. 2022; Hall et al. 2023). This bat species is geographically widespread, highly abundant, and regularly roosts in anthropogenic structures (Davis et al. 1962; Russell et al. 2011; Li and Wilkins 2015), all of which could facilitate human exposure and SARS-CoV-2 spillback. Mexican free-tailed bats also vary widely in their migratory strategies, with some populations undertaking seasonal movements of 100–1000 km between the southwestern United States and Mexico (Villa and Cockrum 1962; Russell et al. 2005), which could facilitate viral dispersal.

**Fig. 1:**
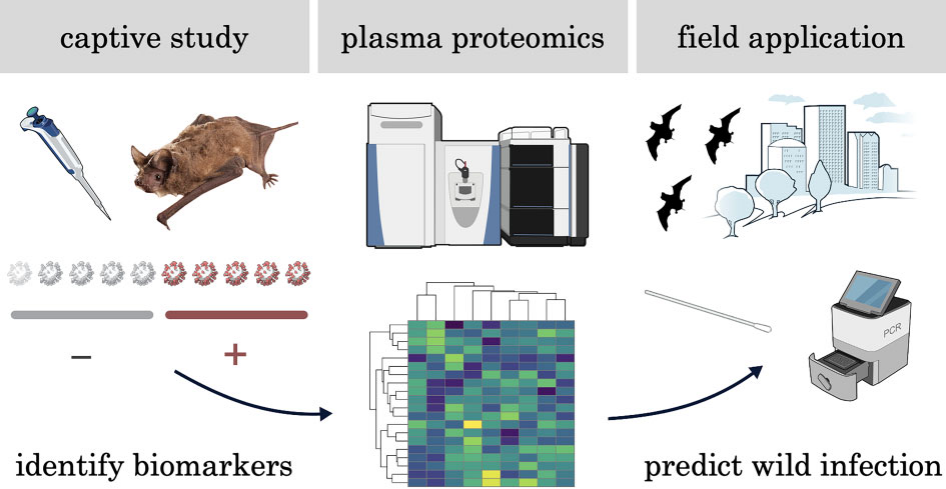
Schematic of the linkages between captive experiments and field studies in the context of SARS-CoV-2 spillback and Mexican free-tailed bats. Viral icons show the number of bats that were uninfected (gray) or susceptible (red) to SARS-CoV-2 challenge, with transparent virions showing bats for which sufficient plasma was not obtainable. Scientific images are from BioArt Source (National Institutes of Health). Mexican free-tailed bat photo credit: Michael Durham.

Here, we capitalize on prior captive challenge studies of Mexican free-tailed bats and ongoing surveys of this species to demonstrate the potential utility of the experimental-to-field pipeline in better understanding and assessing the risk of viral spillback. To first identify candidate biomarkers of SARS-CoV-2 susceptibility, we use advancements in mass spectrometry–based proteomics (Heck and Neely 2020), which can identify hundreds of proteins in the typically low plasma volumes that can be safely collected from small bats (Neely et al. 2021; Becker et al. 2025). Analyzing immune proteins themselves, rather than genes and their expression, also enables us to focus on the functional molecules involved in defense against viral infection. We first analyze plasma proteomes from a small sample of captive bats that differ in susceptibility to SARS-CoV-2, allowing us to identify possible immune mechanisms underlying the probability of infection given exposure or infection progression. We then survey wild Mexican free-tailed bat plasma for those proteins underlying SARS-CoV-2 susceptibility and test if these biomarkers predict naturally occurring CoV infection. We note that prediction here refers to correlative classification ability, and any candidate biomarkers of susceptibility identified here require confirmation with larger, balanced sample sizes as well as functional validation. Figure 1 displays the linkages between data sources and analytics in this experimental-to-field pipeline.

## Methods

### SARS-CoV-2 challenge of captive bats

As reported previously, 10 male Mexican free-tailed bats, originally collected from Williamson County, Texas, in August 2021, were challenged with SARS-CoV-2 (isolate USA-WA1/2020) (Hall et al. 2023). Bats underwent a 30-day quarantine and acclimatization period, and their feces were tested by RT-PCR for CoVs (all samples were negative) prior to experimental challenge. Bats were challenged with 10^5^ TCID_50_ virus both nasally (4 μL) and orally (6 μL) under BSL-3 conditions at the National Wildlife Health Center, US Geological Survey, with inoculum titer verified by qRT-PCR. Nine inoculated bats were co-housed each with a naïve individual to test horizontal transmission, and the remaining inoculated bat was housed individually. One additional pair of co-housed bats served as a sham control. All co-housed and sham control bats were also obtained from Williamson County in August 2021. Bats were sampled every other day over 20 days, with qRT-PCR of RNA extracted from swab samples used to confirm SARS-CoV-2 shedding. Five of the 10 bats excreted detectable SARS-CoV-2 RNA in saliva during the experiment, none of which displayed clinical signs of infection. No co-housed or sham control bats tested positive via qRT-PCR. Bats were euthanized at the end of the experiment (day 20), when blood was collected for plasma. ELISA detected SARS-CoV-2 antibodies in the plasma of only all five infected bats by day 20, and sufficient plasma (2 μL) was available for proteomics for nine challenged bats (only four of the five non-susceptible individuals) and all sham or co-housed, unchallenged bats (*n* = 20 captive samples). We note that the timing of plasma sample collection and lack of pre-inoculation samples limit our ability to robustly differentiate pre-existing host susceptibility or the immune response to SARS-CoV-2.

### Wild bat sampling

As part of a larger study of bat migration, immunity, and infection, we sampled 20 Mexican free-tailed bats at Bracken Cave Preserve in Comal County, Texas, in March 2022 (Becker et al. 2024). Comal County and Williamson County are separated by approximately only 100 km; our samples of these two bat populations are thus likely genetically similar, given low structure observed in previous population genetics analyses of Nearctic populations (Russell et al. 2005; Morales et al. 2016). Bracken Cave hosts tens of millions of Mexican free-tailed bats in the maternity season, representing the largest colony of this species (Wiederholt et al. 2013). As a partially migratory population, the colony declines to approximately 10,000 bats in winter, as the vast majority of individuals migrate in fall to wintering grounds in Mexico (Stepanian and Wainwright 2018). Because migrants begin to arrive back at Bracken Cave in early March, our sampling most likely included primarily overwintering residents and some migratory individuals. Bats were captured upon roost emergence using hand nets and held in individual cloth bags until processing. After recording sex, age, and reproductive status (Morgan et al. 2019), we lanced the propatagial vein with sterile 27G needles and collected blood into serum separator tubes (BD Microtainer) with heparinized capillaries, followed by centrifugation to separate plasma from red blood cells. We also collected oral and rectal swabs (cotton and rayon, respectively; Puritan Medical Products), each preserved in 1 mL DNA/RNA Shield (Zymo Research). Plasma and swab samples were stored in LN2 dry shippers until long-term storage at –80°C at the University of Oklahoma. Sufficient plasma volumes for proteomic analyses were obtained from 15 of the 20 wild bats. All 15 wild bats were adults, with most being female (*n* = 13). Bat sampling was approved by the Institutional Animal Care and Use Committee of the University of Oklahoma (2022–0198) and was authorized by a permit from the Texas Parks and Wildlife Department (SPR-0521–063). All fieldwork was performed according to guidelines for the safe and humane handling of bats published by the American Society of Mammalogists and followed procedures for limiting viral spillback (i.e., use of KN95 face masks, latex and leather gloves, vaccination against and no clinical symptoms of SARS-CoV-2) (Sikes and Gannon 2011; Cook et al. 2022).

### Protein digestion and proteomic profiling

We analyzed both captive (*n* = 20) and wild bat (*n* = 15) plasma samples, which were randomized and processed in a single batch to minimize bias and artifacts. Digestion used S-Trap 96-well mini plates (ProtiFi, 100–300 μg binding capacity), run on a Tecan Resolvex A200 (Tecan Group Ltd., Mannedorf, Switzerland) positive pressure workstation to minimize technical artifacts (Song et al. 2024). To monitor intra-plate variation and qualitatively assess digestion efficiency, LC-MS/MS performance, and downstream search parameters, 2 µL aliquots of NIST SRM 909c Frozen Human Serum (a converted plasma pool) were included at multiple positions across plates. Briefly, 2 µL of plasma (≈ 100 μg protein) was solubilized in 5% SDS, 100 mM TEAB buffer, reduced with 10 mM DL-Dithiothreitol (DTT) at 60°C for 30 min, and alkylated with 20 mM 2-chloroacetamide (CAA) at room temperature for 30 minutes in the dark. Samples were acidified with 12% phosphoric acid and, after mixing, 420 µL binding buffer (90% methanol, 100 mM TEAB, pH 7.55) was added. The entire mix was then transferred to 96-well S-Trap plates and loaded onto the Tecan Resolvex A200. The mix was pushed through the columns with positive pressure, and the columns were washed three times with 300 µL binding buffer, with air flushing between washes. After placing a new collection plate on the retrieved S-Trap plate, trypsin digestion (1:11 trypsin: protein in 110 µL 50 mM TEAB) was performed at 37°C for 1 h, followed by a second 1-h incubation after replenishing with 75 µL 50 mM TEAB. Using the Tecan Resolvex A200, peptides were sequentially eluted with 80 µL 50 mM TEAB, 80 µL 0.2% formic acid, and 80 µL 50% acetonitrile/0.2% formic acid, with air flushing between steps. At the end of the elution protocol, the plate was spun at 2000 g for two minutes to ensure all peptides in solution were eluted into the collector plate through their S-Trap columns. The final eluate was dried under a vacuum centrifuge at low heat before storage at –80°C.

LC-MS/MS was performed at the UC Davis Proteomics Core Facility. Briefly, 200 ng digested protein plus a 1X concentration of iRT peptides (Biognosys) were separated using an EvoSep One UPLC (Evosep Biosystems) (Bache et al. 2018). Liquid chromatography was run using the 100 samples per day method (11.7 minutes sample-to-sample run time) on a PepSep 150 µm x 10 cm C18 column (PepSep) with 1.5 μm particle size (100 Å pores) and a ZDV captive spray emitter (Bruker Daltronics). Mobile phases A and B were water with 0.1% formic acid (v/v) and 100% ACN/water/formic acid (v/v/vol), respectively. Mass spectrometry was run on a hybrid trapped ion mobility spectrometry-quadrupole time of flight mass spectrometer (timsTOF HT, Bruker Daltonics) with a modified nano-electrospray ion source (CaptiveSpray, Bruker Daltonics). The mass spectrometer was operated in diaPASEF mode (Meier et al. 2020). The acquisition scheme for data-independent acquisition (DIA) was 36 25m/z precursor windows (1 Da overlap) per 100 ms TIMS scan. Precursor windows began at 299.5 m/z and continued to 1200.5 m/z. Collision energy was ramped linearly as a function of mobility from 63 eV at 1/K_0_ = 1.3 V × s × cm^2^ to 17 eV at 1/K_0_ = 0.7 V × s × cm^2^. Cycle time was 1.09 s.

We used a *Tadarida brasiliensis* annotation derived from a chromosome-level genome assembly produced by the Bat1K Project (Teeling et al. 2018; Webster et al. 2024). The assembly was generated from a male Mexican free-tailed bat from a different Texas colony. To generate an annotation of coding genes, we integrated transcriptomic data from this bat species with reference-based gene predictions from other mammals. We used long- (i.e., Iso-Seq) and short-read RNA sequencing to generate transcriptomic data from Mexican free-tailed bat brain and testes, analyzed as previously described to produce high-quality open reading frame predictions (Jebb et al. 2020). We classified and filtered transcript-based features such as known and novel canonical and non-canonical splice sites. This removed a small set of transcripts with suboptimal features prior to annotation. For example, fusion transcripts (i.e., chimeras with more than one gene), intra-priming transcripts (i.e., with over 85% or at least 10 contiguous adenines within 20 bp upstream of the 3′ end), low-coverage transcripts (i.e., supporting coding regions by less than three reads), reverse-transcriptase switching predictions (i.e., an exon-skipping pattern due to a retrotranscription gap caused by secondary structures in expressed transcripts), intron retention, and nonsense-mediated decay (premature stop codons) were identified as suboptimal features. Some of these transcripts were later used to annotate untranslated regions. Transcript features used for classification were identified with TAMA-GO (Sim et al. 2020). We also used Tool to infer Orthologs from Genome Alignments (TOGA) to generate predictions, using reference genomes from both human (hg38) and mouse (mm10) (Kirilenko et al. 2023).

We integrated data into a single annotation using EVidenceModeler (Haas et al. 2008), assigning different weights to each set of transcripts as follows: 12 for Iso-Seq transcripts, eight for each TOGA prediction based on human and mouse, and eight for RNA-Seq transcripts. Downstream steps to annotate non-overlapping untranslated regions, enrich the annotation with non-coding RNAs, and assign gene names were performed (Jebb et al. 2020). The highly complete annotation resulted in 52,929 transcripts from 19,889 genes with BUSCO scores for complete genes being 99.2% (Simão et al. 2015). The protein FASTA was then used for DIA.

We used the above annotation and the DIA-NN software suite (2.1.0) to search the raw data directly, without pre-conversion to mzML (Demichev et al. 2020; Kistner et al. 2023). Before searching, we reformatted the protein FASTA to mimic the UniProtKB FASTA format, as recommended for DIA-NN (for use in proteotypicity filtering), allowing DIA-NN to use gene symbols. We did not generate a spectral library before the search, instead allowing DIA-NN to generate this library directly from the FASTA prior to searching. We used default search settings, with the exception of mass accuracy (10.0 ppm) and MS1 accuracy (15.0 ppm), based on recommendations for timsTOF data. The scan window was kept at 0, considering unrelated runs. These settings allowed DIA-NN to evaluate the DIA windowing scheme for each run and set the scans accordingly (all runs were eventually set to six based on the analysis log). Other important default settings to highlight include using trypsin/P with one mis-cleavage, N-term M excision, match between runs, and carbamidomethylation; we also set proteotypicity to “genes.” All settings are given in the Supplemental Material. Our final data matrix included the identified gene groups at a 1% false-discovery rate, which were quantified with proteotypic peptides only. Nine Mexican free-tailed bat proteins did not have human orthologs (1300017J02Rik, Serpina3b, AC004067, IGHV1-34, IGHV1-50, Serpina3c, MBL1, IGHV1-13, Serpinb1a, and Serpina3g), so we used UniProt identifiers from *Mus musculus, Molossus molossus*, and *Myotis davidii*.

### Proteomic data analysis

We stratified our matrix of relative abundances for all identified Mexican free-tailed bat proteins into multiple datasets. Across captive (*n* = 20) and wild (*n* = 15) bat data, we imputed missing values (i.e., zeroes) with half the minimum abundance per protein (Webb-Robertson et al. 2015; Lazar et al. 2016). While multiple methods can be used to replace values below detection limit, our use of minimum abundances should not affect results of the rank-based analyses outlined below. We excluded missing abundance values for descriptive presentation of protein means.

Given limited proteomic data on bats (Hecht‐Höger et al. 2020; Woon et al. 2020; Neely et al. 2021; Becker et al. 2025), we first compared our Mexican free-tailed bat data to our prior data on common vampire bats (*Desmodus rotundus*), in which we used similar pipelines (e.g., S-Trap digestion, DIA-NN) but with the genome annotation for this species (Becker et al. 2022). We used the *eulerr* R package to visualize the shared proteome and proteins unique to each bat species. We then computed the log_10_-transformed mean abundance for each protein per species and used Spearman's correlation to assess similarity in protein abundance between the two bat hosts. For the overall Mexican free-tailed bat data, we also assessed the dynamic range of mean protein abundance, highlighting the top proteins and those unique from the vampire bat proteome. Within our Mexican free-tailed bat dataset, we also quantified the number of proteins shared between captive and wild bats. We again used Spearman’s correlation to assess similarity in protein abundance between captive and wild bats, followed by principal components analysis (PCA) and permutation multivariate analysis of variance (PERMANOVA) with the *vegan* package to test for differential protein composition between bat populations (Dixon 2003).

### Experimental data analysis

We used compositional and rank-based analyses to assess proteomic differences for the nine captive bats challenged with SARS-CoV-2, although results should be interpreted with caution and as preliminary findings given the small sample size (Hanczar et al. 2010; Feng et al. 2017). We first used PCA and PERMANOVA with the *vegan* package to test for differential protein composition as a function of susceptibility (Dixon 2003). To identify differentially abundant proteins between these two groups, we used a two-sided Wilcoxon rank sum test, adjusting *P* values for the inflated false-discovery rate (Benjamini and Hochberg 1995). For each protein, we calculated the log_2_-fold change (LFC) as the difference in mean log_2_-transformed abundance between susceptible and non-susceptible bats. To identify candidate biomarkers of SARS-CoV-2 susceptibility, we next fit receiver operating characteristic (ROC) curves using the *pROC* package (Robin et al. 2011). To measure classifier performance, we estimated the area under the ROC curve (AUC) and 95% confidence intervals (CI) with 5000 bootstraps. For this analysis, we excluded proteins with complete class imbalance prior to imputation of missing values. In cas(F1,33 = 10.9, R2 = 0.25, P < 0.01; Supplementary Fig. S4).es of complete separation (AUC = 1), which can be common in small datasets, we adjusted lower CIs based on sample size and variance ratios (Obuchowski and Lieber 2002). We only considered proteins with AUC ≥ 0.8 to be candidate classifiers (Vicente-Santos et al. 2023), further restricting classifiers to those proteins with a lower CI above 0.5. We also post-hoc estimated the statistical power of each candidate biomarker (Obuchowski et al. 2004). We visualized the matrix of candidate biomarkers with the *pheatmap* package, using scaled and centered log_2_-transformed abundances and Ward’s clustering (Murtagh and Legendre 2014).

We then evaluated up- and down-regulated responses to SARS-CoV-2 susceptibility using gene ontology (GO) and enrichment analysis. We used the *gprofiler2* package to interface with the g:Profiler tool g:GOSt (Raudvere et al. 2019; Kolberg et al. 2020). We included all candidate biomarkers in this analysis, using LFC to determine up- and down-regulated proteins. We ranked proteins via AUC and used incremental enrichment testing, with *P*-values adjusted by the Set Counts and Sizes (SCS) correction. We used *Homo sapiens* as our reference, restricted to GO biological processes. All nine of the bat proteins without human orthologs were poor classifiers (AUC < 0.8), so manual GO and pathway mapping were not required.

### Virus diagnostics

To assess how these candidate biomarkers of SARS-CoV-2 susceptibility could predict CoV risk in the wild, we tested both oral and rectal swabs from the wild bats for CoV infection. We extracted total nucleic acids using the Quick-DNA/RNA Viral Magbead Kit (Zymo Diagnostics), run on an IsoPure 96 platform (Accuris Instruments). Each extraction used 200 μL of DNA/RNA Shield from swabs. We then performed DNase I treatment and RNA cleanup on 10 uL of each extract using the RNA Clean & Concentrator Magbead Kit (Zymo Diagnostics), followed by converting 5 uL RNA into cDNA using LunaScript RT SuperMix (New England BioLabs). We then used semi-nested PCR to amplify an approximately 600 bp segment of the RNA-dependent RNA polymerase (RdRp) gene common to all genera of the *Coronaviridae* (Xiu et al. 2020). All cDNA conversion and PCR were performed in a UV-sterilized PCR workstation (AirClean Systems). Although our group used SARS-CoV-2 (USA-WA1/2020) as a positive control during earlier PCR optimization, we excluded positive controls here to limit risks of contamination.

Positive amplicons were Sanger sequenced at the North Carolina State University Genomic Sciences Laboratory. Resulting forward and reverse sequences were trimmed and cleaned in Geneious (Kearse et al. 2012). Consensus sequences were aligned with reference sequences and top BLASTn hits from GenBank using MUSCLE (Edgar 2004). We then used MrBayes for phylogenetic analysis, run for 1,000,000 generations using a GTR + I + G model in NGPhylogeny.fr (Huelsenbeck and Ronquist 2001; Abadi et al. 2019; Lemoine et al. 2019).

### Wild bat data analysis

We next mined our wild Mexican free-tailed bat proteomes for our candidate susceptibility biomarkers and derived the coefficient of variation (CV) to assess relative heterogeneity in their abundance. We then fit ROC curves for each of these candidate biomarkers in wild bats and measured the ability to classify bats with and without natural CoV infection (i.e., AUC with CIs estimated using 5000 bootstraps). We again excluded proteins with complete class imbalance prior to imputation of missing values; where possible, we stratified analyses by CoV genus.

## Results

### Mexican free-tailed bat proteome

We identified 475 proteins in captive and wild Mexican free-tailed bats. This species shared 62% of the plasma proteome (*n* = 294 proteins) with the vampire bat serum proteome (Supplementary Fig. S1), and abundances of these proteins were positively associated between species (*ρ* = 0.69, *P* < 0.001). The most abundant of these 181 species-specific proteins included immunoglobulin λ–like polypeptide 1 (IGLL1), immunoglobulin heavy variable 8–13 (IGHV8-13), apolipoprotein C-III (APOC3), immunoglobulin heavy constant μ (IGHM), sex hormone-binding globulin (SHBG), fetuin-B (FETUB), complement C4-B (C4B), NLR family member X1 (NLRX1), complement factor H-related protein 3 (CFHR3), and secretoglobin family 1D member 1 (SCGB1D1).

The 475 proteins identified in the Mexican free-tailed bat proteome spanned 4.4 orders of magnitude, with the most abundant proteins including albumin (ALB), apolipoprotein A-I (APOA1), lactotransferrin (LTF), hemopexin (HPX), the above-mentioned IGLL1, APOA2, α-1B-glycoprotein (A1BG), the above-mentioned IGHV8-13, haptoglobin (HP), and C3 complement (C3). The dynamic range and top 20 proteins are displayed in Supplementary Fig. S2.

The proteome of captive bats had 97% of all identified proteins (*n* = 463), whereas that of wild bats only included 84% of all identified proteins (*n* = 398). The most abundant proteins of the captive bats not found in wild bats included the above-mentioned SCGB1D1, neutrophil defensin 1 (DEFA1B), arginine vasopressin (AVP), pentraxin 3 (PTX3), and insulin-like growth factor binding protein 1 (IGFBP1). The most abundant proteins of the wild bats not found in captive bats included hemoglobin subunit γ-2 (HBG2), glycodelin (PAEP), spectrin β chain (SPTB), adenosine deaminase 1 (ADD1), and bifunctional purine biosynthesis protein (ATIC). Captive and wild bats shared 386 proteins (Supplementary Fig. S3), for which abundances were highly positively correlated (*ρ* = 0.87, *P* < 0.001). However, PERMANOVA showed that wild and captive bats did differ in protein composition (*F*_1,33_ = 10.9, *R^2^* = 0.25, *P* < 0.01; Supplementary Fig. S4).

### Proteomic differences with SARS-CoV-2

**Fig. 2:**
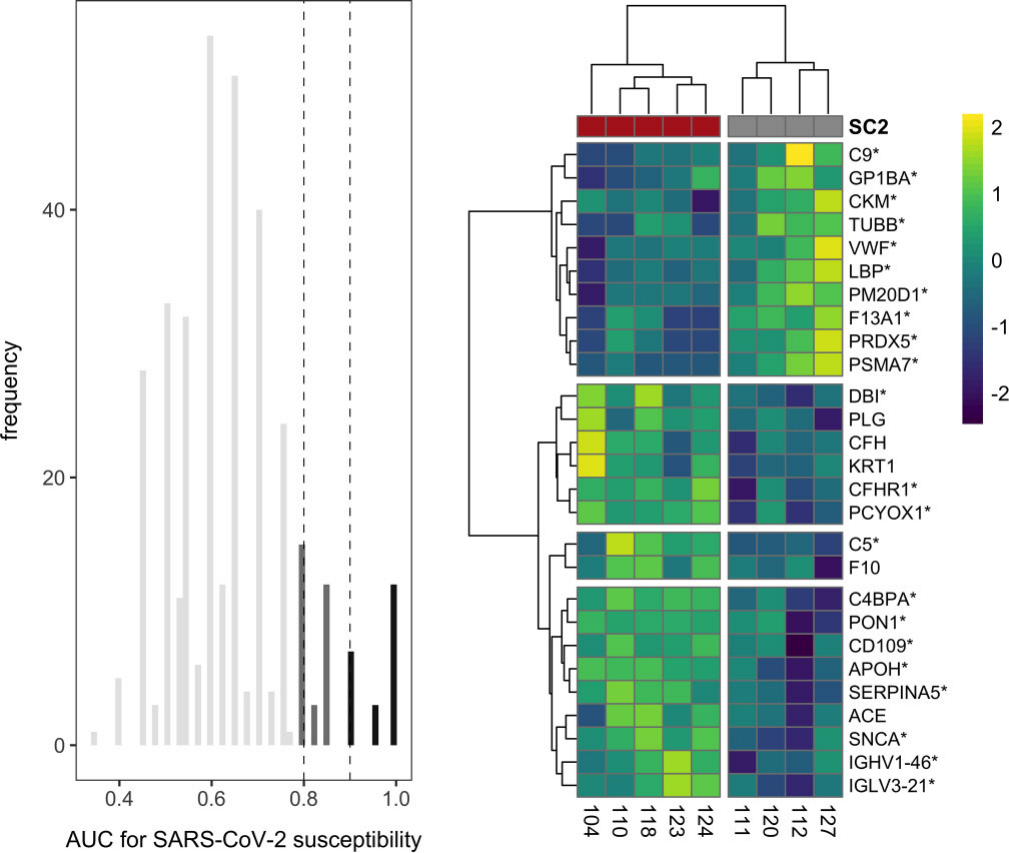
Distribution of AUC for candidate protein classifiers of SARS-CoV-2 susceptibility in captive bats with cutoffs of 0.8 and 0.9 for less-conservative and strict biomarkers, respectively. The heatmap displays the log_10_-transformed abundance for all 27 candidate biomarkers, scaled to a mean of zero. Columns display individuals (susceptibles in red, non-susceptibles in gray), while rows display proteins as gene symbols; those with AUC ≥ 0.9 are marked with an asterisk.

We assessed differential composition, abundance, and classifier ability of 446 proteins found in the small subset of captive bats inoculated with SARS-CoV-2. PERMANOVA found no differences in proteome composition as a function of susceptibility (*F*_1,7_ = 1.21, *R^2^* = 0.15, *P* = 0.30; Supplementary Fig. S5). Similarly, Wilcoxon rank sum tests found no proteins with significant differential abundance between susceptible and non-susceptible bats (Supplementary Fig. S5). However, when we considered the 359 proteins without complete class imbalance prior to imputing zeroes, ROC analyses identified 27 candidate biomarkers of susceptibility (AUC ≥ 0.8, 95% CIs > 0.5; Fig. 2). Most of these proteins (81%) were strong candidate biomarkers (AUC ≥ 0.9): apolipoprotein H (APOH), C4b-binding protein alpha chain (C4BPA), complement C5 (C5), complement factor H-related protein 1 (CFHR1), CD109 antigen (CD109), acyl-CoA-binding protein (DBI), HSC70-interacting protein (F13A1), immunoglobulin λ variable 3–21 (IGLV3-21), peptidase M20 domain containing 1 (PM20D1), serum paraoxonase/arylesterase 1 (PON1), proteasome subunit α type-7 (PSMA7), protein C inhibitor (Serpina5), prenylcysteine oxidase 1 (PCYOX1), α-synuclein (SNCA), von Willebrand factor (VWF), complement component C9 (C9), creatine kinase M-type (CKM), platelet glycoprotein Ib α chain (GP1BA), immunoglobulin heavy variable 1–46 (IGHV1-46), lipopolysaccharide-binding protein (LBP), peroxiredoxin 5 (PRDX5), and tubulin β chain (TUBB). The remaining five candidate biomarkers included angiotensin-converting enzyme (ACE), complement factor H (CFH), keratin 1 (KRT1), plasminogen (PLG), and coagulation factor X (F10). Most of the above proteins (*n* = 17) were positive predictors of susceptibility, with the other 10 proteins serving as negative predictors. Excluding those candidate biomarkers with complete separation (AUC = 1, *n* = 12), most remaining proteins (10/15) had a post-hoc estimated statistical power of at least 60% (three had power of 83%). Despite the small sample size, these 27 candidate biomarkers differentiated the protein phenotypes of SARS-CoV-2 susceptible and non-susceptible bats (Fig. 2).

We next interrogated the up- and down-regulated biological processes underlying SARS-CoV-2 susceptibility using GO terms. Enrichment analyses identified multiple functional protein differences between susceptible and non-susceptible bats after SCS correction (Supplementary Fig. S6). Bats susceptible to SARS-CoV-2 showed downregulation of blood coagulation and protein activation cascade and upregulation of complement activation and humoral immune response.

### Biomarker assessment and CoV infection

**Fig. 3:**
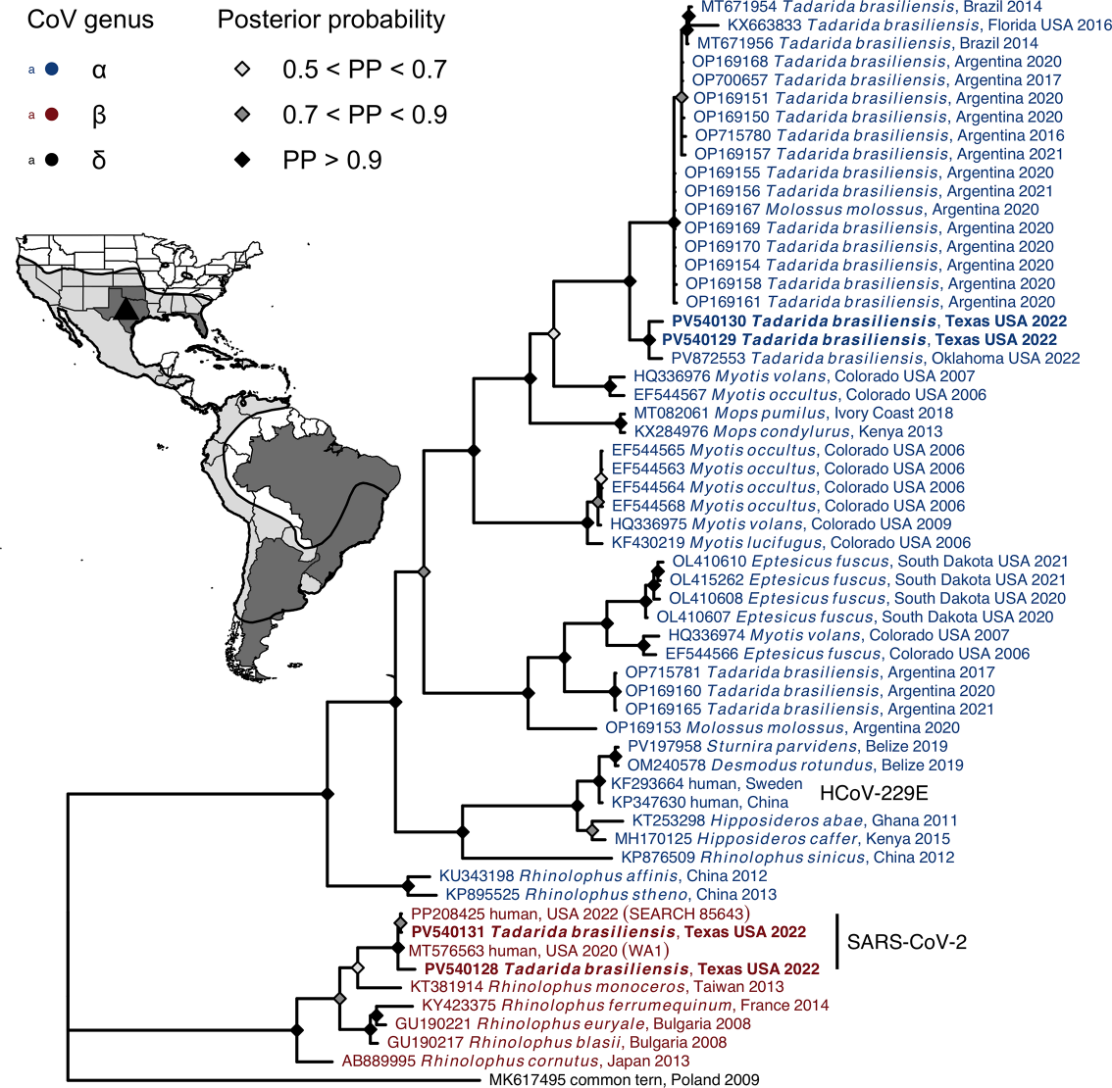
Consensus Bayesian phylogeny of CoV RdRp sequences from wild Mexican free-tailed bats sampled in this study (shown in bold) in relation to other bat- and human-derived sequences. Tips are colored by CoV genus (with an avian δ-CoV used as the outgroup), while nodes are colored by their posterior probability (nodes with less than 50% support are not shown). The inset shows the Mexican free-tailed bat distribution, with gray countries and US states showing sources of CoV-positive bats. The triangle indicates the field site (Bracken Cave Preserve).

We detected CoVs in 20% (95% CI: 8.1–41.6%) of the 20 wild bats: three oral swabs were positive (15%, 95% CI: 5.2–36%), while one rectal swab was positive (5%, 95% CI: 9–23.6%); no bats had both samples test positive (Table S1). Phylogenetic analyses of the RdRp sequences suggested circulation of two distinct α-CoVs (91% similar to one another; Fig. 3), each of which was 86% and 90% similar to CoVs identified in *Tadarida brasiliensis* from Argentina, Brazil, and Florida (e.g., GenBank accessions OP169170, MT671954, and KX663833) (Bonny et al. 2017; Cibulski et al. 2021; Caraballo et al. 2022). We also identified two β-CoV sequences that were 98.6% similar to one another, 98.6–99.4% similar to a 2022 human isolate of SARS-CoV-2 (i.e., PP208425), and 98.6–99.3% similar to the early USA-WA1/2020 isolate (i.e., MT576563). We did not include positive controls in these analyses, and retesting of samples positive for β-CoVs confirmed absence of contamination. Negative controls were also negative. Of the 15 wild bats with proteomic data, only three had CoV data (one α-CoV isolate and two β-CoV isolates).

**Fig. 4:**
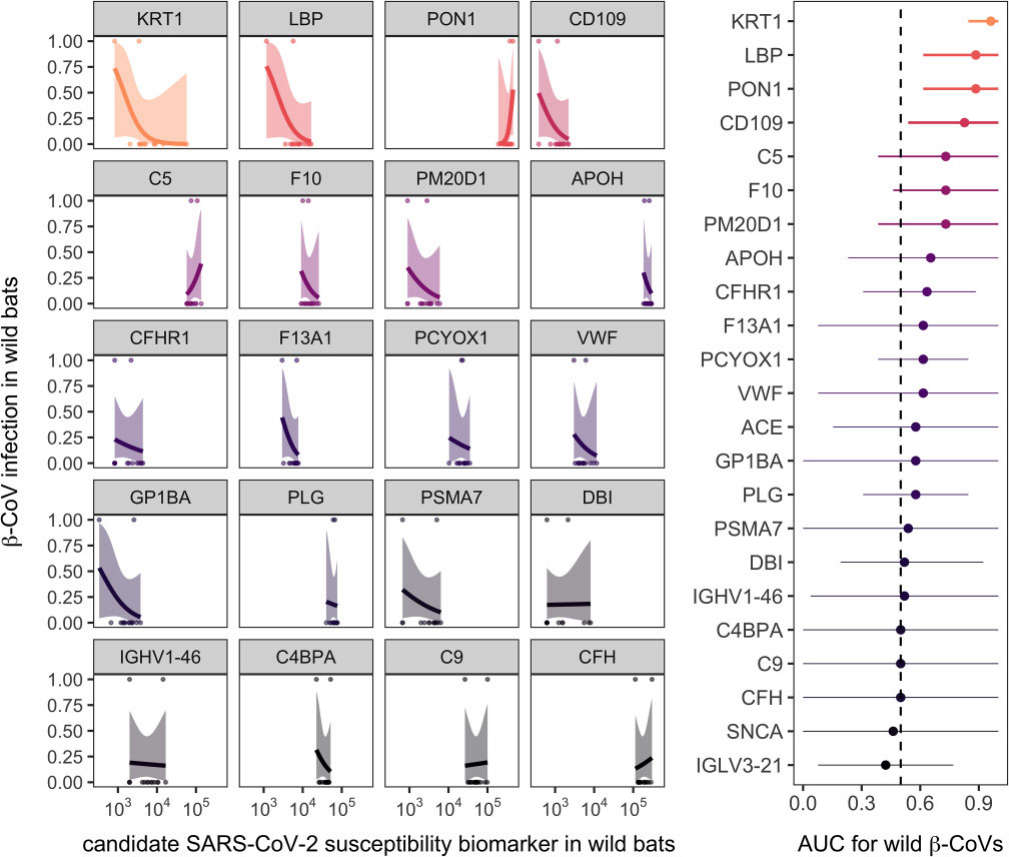
Ability of candidate SARS-CoV-2 susceptibility biomarkers to predict β-CoV infection status in wild Mexican free-tailed bats. For each protein, lines and confidence intervals display univariate GLMs (binary response) fit with mean bias reduction using the *brglm2* package (Kosmidis et al. 2020). Candidate biomarkers are shown in order of decreasing AUC for classifying β-CoV infection, excluding proteins with worse-than-random classifier ability (i.e., AUC < 0.5; SNCA and IGLV321). The right plot shows AUC with 95% CIs (*n* = 5000 bootstraps), with CI thickness corresponding to classifier ability. Data are colored by AUC.

We detected all 27 candidate biomarkers of SARS-CoV-2 susceptibility in this wild bat population, although two proteins were only detected in single individuals (i.e., CKM, TUBB) and were excluded from further analyses. The CV in protein abundance among these 15 wild bats ranged from 16 to 150% per biomarker (x̄ = 41% ± 6%). Given that we only had proteomic data from one bat with an α-CoV, we restricted subsequent analyses to β-CoV positivity. After excluding candidate biomarkers for which proteins were not detected in both β-CoV–positive bats prior to imputation of missing values, AUC for the remaining 23 proteins ranged from 0.42 to 0.96 (x̄ = 0.63 ± 0.03; Fig. 4). KRT1, LBP, PON1, and CD109 were the strongest potential classifiers of β-CoV positivity (AUC > 0.80, 95% CIs > 0.5), while C5, F10, and PM20D1 had moderate classifier ability (AUC > 0.70, but the 95% CI fell below 0.5). All other susceptibility biomarkers had poor predictive ability (i.e., AUC < 0.7 and 95% CIs that fell below 0.5).

## Discussion

Despite increasing attention to the need to integrate observational and experimental approaches to study, predict, and manage the risks of emerging infectious diseases (Plowright et al. 2008; Johnson et al. 2015; Becker and Banerjee 2023), captive studies have not been sufficiently leveraged to inform surveillance of wild populations. Recently, multiple challenge studies have tested susceptibility of North American bats to SARS-CoV-2 (Hall et al. 2021; 2023; 2024; Bosco-Lauth et al. 2022), given the potential risks this novel virus could pose when introduced through spillback into naïve bat communities (Franklin and Bevins 2020; Olival et al. 2020). Here, we capitalized on an experimental assessment of Mexican free-tailed bats, one bat species known to be susceptible to SARS-CoV-2 (Hall et al. 2023), to identify potential biomarkers of susceptibility that could inform monitoring wild bats for CoV risks. Using plasma proteomics, we identified 27 proteins that predicted susceptibility to this virus, and a small subset of these biomarkers classified wild Mexican free-tailed bats infected with CoVs. These findings could facilitate monitoring wild bats for SARS-CoV-2 risks and highlight how captive challenge studies can be integrated with field studies to inform zoonotic and conservation risk.

Our profiling of the Mexican free-tailed bat plasma proteome contributes to ongoing efforts to more holistically characterize and understand within- and between-species variation in bat immunology (Neely et al. 2021; Gonzalez et al. 2024; Becker et al. 2025). Our candidate protein biomarkers differentiated susceptible and non-susceptible bats, underscoring intraspecific variation in immunity. Such heterogeneity could stem from factors including but not limited to age and immunogenetics (e.g., major histocompatibility complex) (Schmid et al. 2023). While the plasma proteomes of captive bats differed from those of wild bats, all 27 experimentally identified candidate biomarkers of SARS-CoV-2 susceptibility were detectable in the field setting. Enrichment analysis suggested that experimentally susceptible bats had downregulation of blood clotting and an upregulation of complement and humoral immunity. In human patients, prior history of complement activation and coagulation disorders predict the severity of COVID-19 (Ramlall et al. 2020; Afzali et al. 2022), such that low baseline blood clotting and higher baseline complement proteins could also predispose North American bats to infection with SARS-CoV-2. In terms of complement, we observed elevated C5, C4BPA, CFHR1, and CFH in susceptible bats. Yet SARS-CoV-2 can also induce activation of the complement cascade (Holter et al. 2020; Ramlall et al. 2020), with COVID-19 patients having elevated C5 levels in particular (Cugno et al. 2020; Holter et al. 2020). We note that plasma from bats challenged with SARS-CoV-2 was collected after 20 days post-infection (Hall et al. 2023), such that we cannot differentiate if the proteomic profiles of susceptible bats indicate pre-challenge conditions or the immune response to this virus. Comparing proteomes before and after SARS-CoV-2 challenge in bats is needed to elucidate whether elevated complement is a risk factor. Such results also suggest more attention is needed to the role of complement in bat immunity (Becker et al. 2019).

To test if these experimental results could inform monitoring viral spillback in field settings, we surveyed wild Mexican free-tailed bats for CoVs. Using established cutoffs for delineating CoV lineages based on the RdRp gene (i.e., 90% similarity) (Anthony et al. 2017), we found two distinct α-CoVs in the Bracken Cave population. One sequence can conservatively be grouped with α-CoVs initially identified in Mexican free-tailed bats in Brazil and Argentina (Cibulski et al. 2021; Caraballo et al. 2022) and in the southeastern US (Bonny et al. 2017). While these other bat populations consist of different migratory strategies and subspecies than those in Texas (Schwartz 1955; McCracken and Gassel 1997), such findings indicate broad circulation across the range of this widely distributed bat. The other α-CoV sequence was only 86% similar and is thus previously undescribed, but sampling of other migratory and partially migratory Mexican free-tailed bat populations in the southwestern US and Mexico could reveal additional circulation and spatial dynamics. Yet in contrast to the likely coevolutionary history of these α-CoVs with their bat host, we identified two potential cases of SARS-CoV-2 spillback. While spillback has been observed in a diverse suite of mammalian hosts (Fagre et al. 2022), observations of SARS-CoV-2 in bats have been rare (Moran et al. 2023; Goldberg et al. 2024), likely owing at least in part to ACE2 receptors that bind to sarbecoviruses with low affinity or overall low expression of ACE2, at least within some bat lineages (Aicher et al. 2022). Finding SARS-CoV-2 in Mexican free-tailed bats is plausible based on clear susceptibility of this host (Bosco-Lauth et al. 2022; Hall et al. 2023). We minimized risks of contamination in the field (e.g., use of KN95 masks and SARS-CoV-2 vaccination) and by excluding positive controls from analyses, and SARS-CoV-2 positives were re-run to confirm results (negative controls were also consistently negative). Further suggesting contamination as unlikely, SARS-CoV-2 was only detected in 5% of samples, and the RdRp sequences were slightly more similar to SARS-CoV-2 circulating in humans at the time of sampling (2022) than to the WA1/2020 isolate used earlier by our group for PCR optimization. Lastly, ongoing CoV surveillance by our group on Mexican free-tailed bats in Oklahoma, using the same protocols, has found only infection with α-CoVs (unpublished; see Fig. 3 for a representative RdRp sequence). Such findings lend further support for spillback of SARS-CoV-2 into Texas Mexican free-tailed bats, although we caution that sequencing other regions of the CoV genome will be needed for conclusive confirmation.

Further work is needed to assess the possible extent of SARS-CoV-2 circulation in this bat species and potential routes of exposure. As we sampled Mexican free-tailed bats in Bracken Cave in March, most individuals were likely overwintering residents rather than spring migrants (Stepanian and Wainwright 2018), suggesting infections were acquired locally. SARS-CoV-2 transmission through wastewater or complex wildlife communities would be plausible, given the large foraging range of this species (e.g., over 40 km) (Davis et al. 1962; Best and Geluso 2003). By contrast to the population monitored here, surveillance of colonies with close interactions with humans, such as those that roost in caves visited by tourists and in bridges (Best and Geluso 2003; Li and Wilkins 2015), would be useful, especially as such roosts may modify Mexican free-tailed bat immunity in ways that increase susceptibility (Allen et al. 2009).

Lastly, we found that only a subset of these candidate SARS-CoV-2 susceptibility biomarkers predicted β-CoV infections in the wild. KRT1, LBP, PON1, and CD109 had the strongest out-of-sample classifier performance. KRT1, LBP, and CD109 were both lower in bats with β-CoV infection, whereas PON1 was higher. Limited work has suggested a role for KRT1 in COVID-19 progression in humans (Li et al. 2024), and keratins are common contaminants in mass spectrometry (Frankenfield et al. 2022). However, KRT1 ranks in the top 40% of proteins in both the bat plasma and human blood proteomes (Uhlén et al. 2019) (Supplementary Fig. S7), such that further inquiry into its role in bat immunity may be warranted, especially given connections between other keratins and the antiviral response in humans (Qu et al. 2025). By contrast, LBP, CD109, and PON1 have all been associated with severity of SARS-CoV-2 infection in humans (Petruk et al. 2020; Gabaldó et al. 2022; Alshathri et al. 2025; de Lima et al. 2025), underscoring potential similarities between the bat and human response to this novel virus. Although further work is needed to identify the role these proteins may play in β-CoV pathogenesis in bats, they could serve as useful targets for creating sensitive, bat-specific parallel reaction monitoring mass spectrometry–based assays (Picotti and Aebersold 2012; Neely et al. 2013). Monitoring shifts in protein abundance could provide early indicators of SARS-CoV-2 susceptibility in wild bats and guide risk assessment, optimally by focusing on colonies in human interfaces and balancing sampling logistics against the challenges posed by large populations of Mexican free-tailed bats.

More generally, our work here highlights useful synergies between experimental and observational approaches to studying infectious diseases in wildlife. This study was especially facilitated by the use of plasma proteomics, as we were able to capitalize on small plasma volumes (i.e., 2 μL) remaining following completion of the captive challenge study and serological testing (Hall et al. 2023). Further, our ability to identify a large number of proteins was facilitated by a high-quality reference genome of the Mexican free-tailed bat and especially complete annotation (i.e., BUSCO completeness of 99.2%). However, the opportunistic nature of this collaboration also means that our analyses were limited by the small sample size of the original experiment as well as class imbalance and confounding when comparing the captive and wild populations (e.g., all captive bats were male, while most wild bats were female). Our small sample size also restricted the ability to statistically correct for these issues, such as the use of analyses that can include confounders (e.g., weighted gene coexpression network analysis), or to apply cross-validation for robust biomarker discovery (Dakna et al. 2010; Pei et al. 2017). Larger and balanced samples will be important to confirm any of the candidate protein biomarkers identified here. At the same time, we note that such limitations are often inherent to studying wild hosts and to *in vivo* infection experiments due to both logistical and ethical concerns (Nishiura et al. 2013; Jia et al. 2020). As such, collaboration between experimental and field-based research would ideally begin at the study design phase, although this may not always be possible in practice. Given these considerations, similar applications could leverage new, ongoing, or completed challenge studies and include, but are not limited to, using within-host antibody kinetics to estimate time of viral exposure (Pepin et al. 2017) and applying experimental biomarkers to identify especially competent hosts in the wild (Burgan et al. 2018). We encourage field-based researchers and those undertaking captive challenge studies to seek out such mutual collaborative opportunities to better predict and manage emerging disease risks.

## Supplementary Material

icaf148_Supplemental_File

## Data Availability

Proteomic data from captive and wild Mexican free-tailed bats, the Mexican free-tailed bat protein FASTA, and DIA-NN settings are available on MassIVE (MSV000098424). The genome assembly for this bat species is available in the European Nucleotide Archive (PRJNA972445). CoV RdRp sequences from wild bats are available on GenBank (PV540128–31).

## References

[bib1] Abadi S , Azouri D, Pupko T, Mayrose I. 2019. Model selection may not be a mandatory step for phylogeny reconstruction. Nat Commun. 10:934. 30804347 10.1038/s41467-019-08822-w PMC6389923

[bib2] Afzali B , Noris M, Lambrecht BN, Kemper C. 2022. The state of complement in COVID-19. Nat Rev Immunol. 22:77–84. 34912108 10.1038/s41577-021-00665-1 PMC8672651

[bib3] Aicher S-M , Streicher F, Chazal M, Planas D, Luo D, Buchrieser J, Nemcova M, Seidlova V, Zukal J, Serra-Cobo J et al. 2022. Species-specific molecular barriers to SARS-CoV-2 replication in bat cells. J Virol. 96:e0060822. 35862713 10.1128/jvi.00608-22 PMC9327701

[bib4] Allen LC , Turmelle AS, Mendonça MT, Navara KJ, Kunz TH, McCracken GF. 2009. Roosting ecology and variation in adaptive and innate immune system function in the Brazilian free-tailed bat (*Tadarida brasiliensis*). J Comp Physiol B. 179:315–23. 19002470 10.1007/s00360-008-0315-3 PMC7087743

[bib5] Alshathri A , Bindayel I, Alabdullatif W, Alhijji A, Albarrag A. 2025. Lipopolysaccharide-binding protein (LBP) and inflammatory biomarkers in SARS-CoV-2 hospitalized patients. J Clin Med. 14:4075. 40565820 10.3390/jcm14124075 PMC12194322

[bib6] Anthony SJ , Johnson CK, Greig DJ, Kramer S, Che X, Wells H, Hicks AL, Joly DO, Wolfe ND, Daszak P et al. 2017. Global patterns in coronavirus diversity. Virus Evol. 3:vex012. 28630747 10.1093/ve/vex012 PMC5467638

[bib7] Bache N , Geyer PE, Bekker-Jensen DB, Hoerning O, Falkenby L, Treit PV, Mann. 2018. A novel LC system embeds analytes in pre-formed gradients for rapid, ultra-robust proteomics. Mol Cell Proteomics. 17:2284–96. 30104208 10.1074/mcp.TIR118.000853 PMC6210218

[bib8] Becker DJ , Banerjee A. 2023. Coupling field and laboratory studies of immunity and infection in zoonotic hosts. Lancet Microbe. 4:e285–e287. 36878243 10.1016/S2666-5247(23)00032-0 PMC9984197

[bib9] Becker DJ , Czirják GÁ, Rynda-Apple A, Plowright RK. 2019. Handling stress and sample storage are associated with weaker complement-mediated bactericidal ability in birds but not bats. Physiol Biochem Zool. 92:37–48. 30481115 10.1086/701069 PMC7241871

[bib10] Becker DJ , Dyer KE, Lock LR, Olbrys BL, Pladas SA, Sukhadia AA, Demory B, Nunes Batista JM, Pineda M, Simmons NB et al. 2024. Geographically widespread and novel hemotropic mycoplasmas and bartonellae in Mexican free-tailed bats and sympatric North American bat species. mSphere. 10:e0011624. 39660872 10.1128/msphere.00116-24 PMC11774037

[bib11] Becker DJ , Lei G-S, Janech MG, Bland AM, Fenton MB, Simmons NB, Relich RF, Neely BA. 2022. Serum proteomics identifies immune pathways and candidate biomarkers of coronavirus infection in wild vampire bats. Front Virol. 2.862961

[bib12] Becker DJ , Vicente-Santos A, Reers A, Ansil BR, O'Shea M, Cummings C, Roistacher A, Quintela-Tizon R, Pereira M, Rosen J et al. 2025. Diverse hosts, diverse immune systems: evolutionary variation in bat immunology.Annals of the New York Academy of Sciences. 10.1111/nyas.15395 PMC12412728 40607689

[bib13] Benjamini Y , Hochberg Y. 1995. Controlling the false discovery rate: a practical and powerful approach to multiple testing. J R Stat Soc Series B Stat Methodol. 57:289–300.

[bib14] Best TL , Geluso KN. 2003. Summer foraging range of Mexican free-tailed bats (*Tadarida brasiliensis mexicana*) from Carlsbad Cavern, New Mexico. The Southwestern Naturalist. 48:590–6.

[bib15] Boni MF , Lemey P, Jiang X, Lam TT-Y, Perry BW, Castoe TA, Rambaut A, Robertson DL. 2020. Evolutionary origins of the SARS-CoV-2 sarbecovirus lineage responsible for the COVID-19 pandemic. Nat Microbiol. 5:1408–17. 32724171 10.1038/s41564-020-0771-4

[bib16] Bonny TS , Driver J, Paisie T, Salemi M, Morris J, Shender LA, Smith L, Enloe C, Oxenrider KJ, Gore J et al. 2017. Detection of alphacoronavirus vRNA in the feces of Brazilian free-tailed bats (*Tadarida brasiliensis*) from a colony in Florida, USA. Diseases. 5:7. 28933360 10.3390/diseases5010007 PMC5456339

[bib17] Bosco-Lauth AM , Porter SM, Fox KA, Wood ME, Neubaum D, Quilici M. 2022. Experimental infection of Brazilian free-tailed bats (*Tadarida brasiliensis*) with two strains of SARS-CoV-2. Viruses. 14:1809. 36016431 10.3390/v14081809 PMC9412320

[bib18] Burgan SC , Gervasi SS, Martin LB. 2018. Parasite tolerance and host competence in avian host defense to West Nile virus. EcoHealth. 15:360–71. 29569179 10.1007/s10393-018-1332-7

[bib19] Caraballo DA , Sabio MS, Colombo VC, Piccirilli MG, Vico L, Hirmas Riade SM, Campos J, Martínez G, Beltrán F, Baumeister E et al. 2022. The role of Molossidae and Vespertilionidae in shaping the diversity of alphacoronaviruses in the Americas. Microbiol Spectr. 10:e0314322. 36222689 10.1128/spectrum.03143-22 PMC9769993

[bib20] Cerdà-Cuéllar M , Moré E, Ayats T, Aguilera M, Muñoz-González S, Antilles N, Ryan PG, González-Solís J. 2019. Do humans spread zoonotic enteric bacteria in Antarctica?. Sci Total Environ. 654:190–6. 30445320 10.1016/j.scitotenv.2018.10.272

[bib21] Cheng TL , Reichard JD, Coleman JTH, Weller TJ, Thogmartin WE, Reichert BE, Bennett AB, Broders HG, Campbell J, Etchison K et al. 2021. The scope and severity of white-nose syndrome on hibernating bats in North America. Conserv Biol. 35:1586–97. 33877716 10.1111/cobi.13739 PMC8518069

[bib22] Cibulski SP , de Sales Lima FE, Teixeira TF, Varela APM, Scheffer CM, Mayer FQ, Witt AA, Roehe PM. 2021. Detection of multiple viruses in oropharyngeal samples from Brazilian free-tailed bats (*Tadarida brasiliensis*) using viral metagenomics. Arch Virol. 166:207–12. 33047159 10.1007/s00705-020-04825-x PMC7549734

[bib23] Cohen LE , Fagre AC, Chen B, Carlson CJ, Becker DJ. 2023. Coronavirus sampling and surveillance in bats from 1996-2019: a systematic review and meta-analysis. Nat Microbiol. 8:1176–86. 37231088 10.1038/s41564-023-01375-1 PMC10234814

[bib24] Cook JD , Campbell Grant EH, Coleman JTH, Sleeman JM, Runge MC. 2022. Evaluating the risk of SARS-CoV-2 transmission to bats in the context of wildlife research, rehabilitation, and control. Wildl Soc Bull. 46:e1262.

[bib25] Cugno M , Meroni PL, Gualtierotti R, Griffini S, Grovetti E, Torri A, Panigada M, Aliberti S, Blasi F, Tedesco F et al. 2020. Complement activation in patients with COVID-19: a novel therapeutic target. J Allergy Clin Immunol. 146:215–7. 32417135 10.1016/j.jaci.2020.05.006 PMC7224678

[bib26] Dakna M , Harris K, Kalousis A, Carpentier S, Kolch W, Schanstra JP, Haubitz M, Vlahou A, Mischak H, Girolami M. 2010. Addressing the challenge of defining valid proteomic biomarkers and classifiers. BMC Bioinf. 11:594. 10.1186/1471-2105-11-594 PMC3017845 21208396

[bib27] Davis RB , Herreid CF, Short HL. 1962. Mexican free-tailed bats in Texas. Ecol Monogr. 32:311–46.

[bib28] Davy CM , Donaldson ME, Subudhi S, Rapin N, Warnecke L, Turner JM, Bollinger TK, Kyle CJ, Dorville NAS-Y, Kunkel EL et al. 2018. White-nose syndrome is associated with increased replication of a naturally persisting coronaviruses in bats. Sci Rep. 8:15508. 30341341 10.1038/s41598-018-33975-x PMC6195612

[bib29] de Lima IL , Cataldi TR, Brites C, Labate MTV, Vaz SN, Deminco F, da Cunha GS, Labate CA, Eberlin MN. 2025. 4D-DIA proteomics uncovers new insights into host salivary response following SARS-CoV-2 Omicron infection. J Proteome Res. 24:499–514. 39803891 10.1021/acs.jproteome.4c00630 PMC11812090

[bib30] Demichev V , Messner CB, Vernardis SI, Lilley KS, Ralser M. 2020. DIA-NN: neural networks and interference correction enable deep proteome coverage in high throughput. Nat Methods. 17:41–4. 31768060 10.1038/s41592-019-0638-x PMC6949130

[bib31] Dixon P . 2003. VEGAN, a package of R functions for community ecology. J Veg Sci. 14:927–30.

[bib32] Eckstrand CD , Baldwin TJ, Rood KA, Clayton MJ, Lott JK, Wolking RM, Bradway DS, Baszler T. 2021. An outbreak of SARS-CoV-2 with high mortality in mink (*Neovison vison*) on multiple Utah farms. PLoS Pathog. 17:e1009952. 34767598 10.1371/journal.ppat.1009952 PMC8589170

[bib33] Edgar RC . 2004. MUSCLE: multiple sequence alignment with high accuracy and high throughput. Nucleic Acids Res. 32:1792–7. 15034147 10.1093/nar/gkh340 PMC390337

[bib34] Epstein JH , Price JT. 2009. The significant but understudied impact of pathogen transmission from humans to animals: impact of pathogen transmission from humans to animals. Mt Sinai J Med. 76:448–55. 19787650 10.1002/msj.20140 PMC7168516

[bib35] Fagre AC , Cohen LE, Eskew EA, Farrell M, Glennon E, Joseph MB, Frank HK, Ryan SJ, Carlson CJ, Albery GF. 2022. Assessing the risk of human-to-wildlife pathogen transmission for conservation and public health. Ecol Lett. 25:1534–49. 35318793 10.1111/ele.14003 PMC9313783

[bib36] Feng A , Bevins S, Chandler J, DeLiberto TJ, Ghai R, Lantz K, Lenoch J, Retchless A, Shriner S, Tang CY et al. 2023. Transmission of SARS-CoV-2 in free-ranging white-tailed deer in the United States. Nat Commun. 14:4078. 37429851 10.1038/s41467-023-39782-x PMC10333304

[bib37] Feng D , Cortese G, Baumgartner R. 2017. A comparison of confidence/credible interval methods for the area under the ROC curve for continuous diagnostic tests with small sample size. Stat Methods Med Res. 26:2603–21. 26323286 10.1177/0962280215602040

[bib38] Frankenfield AM , Ni J, Ahmed M, Hao L. 2022. Protein contaminants matter: building universal protein contaminant libraries for DDA and DIA proteomics. J Proteome Res. 21:2104–13. 35793413 10.1021/acs.jproteome.2c00145 PMC10040255

[bib39] Franklin AB , Bevins SN. 2020. Spillover of SARS-CoV-2 into novel wild hosts in North America: a conceptual model for perpetuation of the pathogen. Sci Total Environ. 733:139358. 32416535 10.1016/j.scitotenv.2020.139358 PMC7214292

[bib40] Frick WF , Puechmaille SJ, Hoyt JR, Nickel BA, Langwig KE, Foster JT, Barlow KE, Bartonička T, Feller D, Haarsma A-J et al. 2015. Disease alters macroecological patterns of North American bats. Glob Ecol Biogeogr. 24:741–9.

[bib41] Gabaldó X , Juanpere M, Castañé H, Rodríguez-Tomàs E, López-Azcona AF, Baiges-Gaya G, Castro L, Valverde-Díaz E, Muñoz-Blázquez A, Giménez-Cuenca L et al. 2022. Usefulness of the measurement of serum paraoxonase-1 arylesterase activity in the diagnoses of COVID-19. Biomolecules. 12:879. 35883435 10.3390/biom12070879 PMC9312761

[bib42] Ge X-Y , Li J-L, Yang X-L, Chmura AA, Zhu G, Epstein JH, Mazet JK, Hu B, Zhang W, Peng C et al. 2013. Isolation and characterization of a bat SARS-like coronavirus that uses the ACE2 receptor. Nature. 503:535–8. 24172901 10.1038/nature12711 PMC5389864

[bib43] Goldberg AR , Langwig KE, Brown KL, Marano JM, Rai P, King KM, Sharp AK, Ceci A, Kailing CD, Kailing MJ et al. 2024. Widespread exposure to SARS-CoV-2 in wildlife communities. Nat Commun. 15:6210. 39075057 10.1038/s41467-024-49891-w PMC11286844

[bib44] Gonzalez V , Hurtado-Monzón AM, O'Krafka S, Mühlberger E, Letko M, Frank HK, Laing ED, Phelps KL, Becker DJ, Munster VJ et al. 2024. Studying bats using a One Health lens: bridging the gap between bat virology and disease ecology. J Virol. 98:e0145324. 39499009 10.1128/jvi.01453-24 PMC11650978

[bib45] Haas BJ , Salzberg SL, Zhu W, Pertea M, Allen JE, Orvis J, White O, Buell CR, Wortman JR. 2008. Automated eukaryotic gene structure annotation using EVidenceModeler and the Program to assemble spliced alignments. Genome Biol. 9:R7. 18190707 10.1186/gb-2008-9-1-r7 PMC2395244

[bib46] Hall JS , Hofmeister E, Ip HS, Nashold SW, Leon AE, Malavé CM, Falendysz EA, Rocke TE, Carossino M, Balasuriya U et al. 2023. Experimental infection of Mexican free-tailed bats (*Tadarida brasiliensis*) with SARS-CoV-2. mSphere. 8:e0026322. 36598226 10.1128/msphere.00263-22 PMC9942575

[bib47] Hall JS , Knowles S, Nashold SW, Ip HS, Leon AE, Rocke T, Keller S, Carossino M, Balasuriya U, Hofmeister E. 2021. Experimental challenge of a North American bat species, big brown bat (*Eptesicus fuscus*), with SARS-CoV-2. Transbound Emerg Dis. 68:3443–52. 33295095 10.1111/tbed.13949

[bib48] Hall JS , Nashold S, Hofmeister E, Leon AE, Falendysz EA, Ip HS, Malavé CM, Rocke TE, Carossino M, Balasuriya U et al. 2024. Little brown bats (*Myotis lucifugus*) are resistant to SARS-CoV-2 infection. J Wildl Dis. 60:924–30. 39053909 10.7589/JWD-D-23-00114

[bib49] Hammer AS , Quaade ML, Rasmussen TB, Fonager J, Rasmussen M, Mundbjerg K, Lohse L, Strandbygaard B, Jørgensen CS, Alfaro-Núñez A et al. 2021. SARS-CoV-2 transmission between Mink (*Neovison vison*) and humans, Denmark. Emerg Infect Dis. 27:547–51. 33207152 10.3201/eid2702.203794 PMC7853580

[bib50] Hanczar B , Hua J, Sima C, Weinstein J, Bittner M, Dougherty ER. 2010. Small-sample precision of ROC-related estimates. Bioinformatics. 26:822–30. 20130029 10.1093/bioinformatics/btq037

[bib51] Harvey JA , Mullinax JM, Runge MC, Prosser DJ. 2023. The changing dynamics of highly pathogenic avian influenza H5N1: next steps for management & science in North America. Biol Conserv. 282:110041.

[bib52] Hecht-Höger AM , Braun BC, Krause E, Meschede A, Krahe R, Voigt CC, Greenwood AD, Czirják GÁ. 2020. Plasma proteomic profiles differ between European and North American myotid bats colonized by *Pseudogymnoascus destructans*. Mol Ecol. 29:1745–55. 32279365 10.1111/mec.15437

[bib53] Heck M , Neely BA. 2020. Proteomics in non-model organisms: a new analytical frontier. J Proteome Res. 19:3595–606. 32786681 10.1021/acs.jproteome.0c00448 PMC7874939

[bib54] Hernández-Aguilar I , Lorenzo C, Santos-Moreno A, Naranjo EJ, Navarrete-Gutiérrez D. 2021. Coronaviruses in bats: a review for the Americas. Viruses. 13:1226. 34201926 10.3390/v13071226 PMC8310043

[bib55] Holter JC , Pischke SE, de Boer E, Lind A, Jenum S, Holten AR, Tonby K, Barratt-Due A, Sokolova M, Schjalm C et al. 2020. Systemic complement activation is associated with respiratory failure in COVID-19 hospitalized patients. Proc Natl Acad Sci USA. 117:25018–25. 32943538 10.1073/pnas.2010540117 PMC7547220

[bib56] Hooton LA , Adams AA, Cameron A, Fraser EE, Hale L, Kingston S, Fenton MB, McGuire L, Stukenholtz EE, Davy C. 2023. Effects of bat white-nose syndrome on hibernation and swarming aggregations of bats in Ontario. Can. J. Zool. 101:886–95.,.

[bib57] Hu B , Zeng L-P, Yang X-L, Ge X-Y, Zhang W, Li B, Xie J-Z, Shen X-R, Zhang Y-Z, Wang N et al. 2017. Discovery of a rich gene pool of bat SARS-related coronaviruses provides new insights into the origin of SARS coronavirus. PLoS Pathog. 13:e1006698. 29190287 10.1371/journal.ppat.1006698 PMC5708621

[bib58] Huelsenbeck JP , Ronquist F. 2001. MRBAYES: bayesian inference of phylogenetic trees. Bioinformatics. 17:754–5. 11524383 10.1093/bioinformatics/17.8.754

[bib59] Jebb D , Huang Z, Pippel M, Hughes GM, Lavrichenko K, Devanna P, Winkler S, Jermiin LS, Skirmuntt EC, Katzourakis A et al. 2020. Six reference-quality genomes reveal evolution of bat adaptations. Nature. 583:578–84. 32699395 10.1038/s41586-020-2486-3 PMC8075899

[bib60] Jia B , Colling A, Stallknecht DE, Blehert D, Bingham J, Crossley B, Eagles D, Gardner IA. 2020. Validation of laboratory tests for infectious diseases in wild mammals: review and recommendations. J Vet Diagn Invest. 32:776–92. 32468923 10.1177/1040638720920346 PMC7649530

[bib61] Johnson PTJ , Ostfeld RS, Keesing F. 2015. Frontiers in research on biodiversity and disease. Ecol Lett. 18:1119–33. 26261049 10.1111/ele.12479 PMC4860816

[bib62] Kearse M , Moir R, Wilson A, Stones-Havas S, Cheung M, Sturrock S, Buxton S, Cooper A, Markowitz S, Duran C et al. 2012. Geneious Basic: an integrated and extendable desktop software platform for the organization and analysis of sequence data. Bioinformatics. 28:1647–9. 22543367 10.1093/bioinformatics/bts199 PMC3371832

[bib63] Kirilenko BM , Munegowda C, Osipova E, Jebb D, Sharma V, Blumer M, Morales AE, Ahmed A-W, Kontopoulos D-G, Hilgers L et al. 2023. Integrating gene annotation with orthology inference at scale. Science. 380:eabn3107. 37104600 10.1126/science.abn3107 PMC10193443

[bib64] Kistner F , Grossmann JL, Sinn LR, Demichev V. 2023. QuantUMS: uncertainty minimisation enables confident quantification in proteomics. bioRxiv.

[bib65] Kolberg L , Raudvere U, Kuzmin I, Vilo J, Peterson H. 2020. gprofiler2—an R package for gene list functional enrichment analysis and namespace conversion toolset g:profiler. F1000Res. 9:709. 10.12688/f1000research.24956.1 PMC7859841 33564394

[bib66] Kosmidis I , Kenne Pagui EC, Sartori N. 2020. Mean and median bias reduction in generalized linear models. Stat Comput. 30:43–59.

[bib67] Kuchipudi SV , Surendran-Nair M, Ruden RM, Yon M, Nissly RH, Vandegrift KJ, Nelli RK, Li L, Jayarao BM, Maranas CD et al. 2022. Multiple spillovers from humans and onward transmission of SARS-CoV-2 in white-tailed deer. Proc Natl Acad Sci USA. 119:e2121644119. 35078920 10.1073/pnas.2121644119 PMC8833191

[bib68] Lau SKP , Woo PCY, Li KSM, Huang Y, Tsoi H-W, Wong BHL, Wong SSY, Leung S-Y, Chan K-H, Yuen K-Y. 2005. Severe acute respiratory syndrome coronavirus-like virus in Chinese horseshoe bats. Proc Natl Acad Sci USA. 102:14040–5. 16169905 10.1073/pnas.0506735102 PMC1236580

[bib69] Lazar C , Gatto L, Ferro M, Bruley C, Burger T. 2016. Accounting for the multiple natures of missing values in label-free quantitative proteomics data sets to compare imputation strategies. J Proteome Res. 15:1116–25. 26906401 10.1021/acs.jproteome.5b00981

[bib70] Lemoine F , Correia D, Lefort V, Doppelt-Azeroual O, Mareuil F, Cohen-Boulakia S, Gascuel O. 2019. NGPhylogeny.Fr: new generation phylogenetic services for non-specialists. Nucleic Acids Res. 47:W260–65. 31028399 10.1093/nar/gkz303 PMC6602494

[bib71] Li H , Wilkins KT. 2015. Selection of building roosts by Mexican free-tailed bats (*Tadarida brasiliensis*) in an urban area. Acta Chiropt. 17:321–30.

[bib72] Li X , Ding G, Li S, Liu C, Zheng X, Luo J, He S, Zeng F, Huang X, Zeng F. 2024. Proteomic characteristics of the treatment trajectory of patients with COVID-19. Arch Virol. 169:84. 38532129 10.1007/s00705-024-05991-y

[bib73] Maestri R , Perez-Lamarque B, Zhukova A, Morlon H. 2024. Recent evolutionary origin and localized diversity hotspots of mammalian coronaviruses. eLife. 13:RP91745. 39196812 10.7554/eLife.91745 PMC11357359

[bib74] McAloose D , Laverack M, Wang L, Killian ML, Caserta LC, Yuan F, Mitchell PK, Queen K, Mauldin MR, Cronk BD et al. 2020. From people to *Panthera*: natural SARS-CoV-2 infection in tigers and lions at the Bronx Zoo. mBio. 11:e02220–20. 33051368 10.1128/mBio.02220-20 PMC7554670

[bib75] McCracken GF , Gassel MF. 1997. Genetic structure in migratory and nonmigratory populations of Brazilian free-tailed bats. J Mammal. 78:348–57.

[bib76] Meadows AJ , Stephenson N, Madhav NK, Oppenheim B. 2023. Historical trends demonstrate a pattern of increasingly frequent and severe spillover events of high-consequence zoonotic viruses. BMJ Glob Health. 8:e012026. 10.1136/bmjgh-2023-012026 PMC10626885 37918874

[bib77] Meier F , Brunner A-D, Frank M, Ha A, Bludau I, Voytik E, Kaspar-Schoenefeld S, Lubeck M, Raether O, Bache N et al. 2020. diaPASEF: parallel accumulation-serial fragmentation combined with data-independent acquisition. Nat Methods. 17:1229–36. 33257825 10.1038/s41592-020-00998-0

[bib78] Morales A , Villalobos F, Velazco PM, Simmons NB, Piñero D. 2016. Environmental niche drives genetic and morphometric structure in a widespread bat. J Biogeogr. 43:1057–68.

[bib79] Morales AE , Dong Y, Brown T, Baid K, Kontopoulos D-G, Gonzalez V, Huang Z, Ahmed A-W, Bhuinya A, Hilgers L et al. 2025. Bat genomes illuminate adaptations to viral tolerance and disease resistance. Nature. 638:1–10. 10.1038/s41586-024-08471-0 PMC11821529 39880942

[bib80] Moran ML , Boyd W, De La Cruz JL, Bertke AS. 2023. Oral sampling of little brown bat (*Myotis lucifugus*) maternity colonies for SARS-CoV-2 in the northeast and mid-Atlantic, USA. Animals. 13:550. 36830336 10.3390/ani13040550 PMC9951713

[bib81] Morgan CN , Ammerman LK, Demere KD, Doty JB, Nakazawa YJ, Mauldin MR. 2019. Field identification key and guide for bats of the United States of America. Occas Pap Tex Tech Univ Mus. 360:360. 31148880 PMC6537616

[bib82] Murtagh F , Legendre P. 2014. Ward's hierarchical agglomerative clustering method: which algorithms implement ward's criterion?. J Classif. 31:274–95.

[bib83] Neely BA , Carlin KP, Arthur JM, McFee WE, Janech MG. 2013. Ratiometric measurements of adiponectin by mass spectrometry in bottlenose dolphins (*Tursiops truncatus*) with iron overload reveal an association with insulin resistance and glucagon. Front Endocrinol. 4:132. 10.3389/fendo.2013.00132 PMC3778387 24065958

[bib84] Neely BA , Janech MG, Fenton MB, Simmons NB, Bland AM, Becker DJ. 2021. Surveying the vampire bat (*Desmodus rotundus*) serum proteome: a resource for identifying immunological proteins and detecting pathogens. J Proteome Res. 20:2547–59. 33840197 10.1021/acs.jproteome.0c00995 PMC9812275

[bib85] Negrey JD , Reddy RB, Scully EJ, Phillips-Garcia S, Owens LA, Langergraber KE, Mitani JC, Emery Thompson M, Wrangham RW, Muller MN et al. 2019. Simultaneous outbreaks of respiratory disease in wild chimpanzees caused by distinct viruses of human origin. Emerg Microbes Infect. 8:139–49. 30866768 10.1080/22221751.2018.1563456 PMC6455141

[bib86] Nishiura H , Yen H-L, Cowling BJ. 2013. Sample size considerations for one-to-one animal transmission studies of the influenza A viruses. PLoS One. 8:e55358. 23383167 10.1371/journal.pone.0055358 PMC3561278

[bib87] Obuchowski NA , Lieber ML. 2002. Confidence bounds when the estimated ROC area is 1.01. Acad Radiol. 9:526–30. 12458878 10.1016/s1076-6332(03)80329-x

[bib88] Obuchowski NA , Lieber ML, Wians FH Jr. 2004. ROC curves in clinical chemistry: uses, misuses, and possible solutions. Clin Chem. 50:1118–25. 15142978 10.1373/clinchem.2004.031823

[bib89] Olival KJ , Cryan PM, Amman BR, Baric RS, Blehert DS, Brook CE, Calisher CH, Castle KT, Coleman JTH, Daszak P et al. 2020. Possibility for reverse zoonotic transmission of SARS-CoV-2 to free-ranging wildlife: a case study of bats. PLoS Pathog. 16:e1008758. 32881980 10.1371/journal.ppat.1008758 PMC7470399

[bib90] Oreshkova N , Molenaar RJ, Vreman S, Harders F, Oude Munnink BB, Hakze-van der Honing RW, Gerhards N, Tolsma P, Bouwstra R, Sikkema RS et al. 2020. SARS-CoV-2 infection in farmed minks, the Netherlands, April and May 2020. Euro Surveill. 25:2001005. 32553059 10.2807/1560-7917.ES.2020.25.23.2001005 PMC7403642

[bib91] Pei G , Chen L, Zhang W. 2017. WGCNA application to proteomic and metabolomic data analysis. Methods Enzymol. 585:135–58. 28109426 10.1016/bs.mie.2016.09.016

[bib92] Pepin KM , Kay SL, Golas BD, Shriner SS, Gilbert AT, Miller RS, Graham AL, Riley S, Cross PC, Samuel MD et al. 2017. Inferring infection hazard in wildlife populations by linking data across individual and population scales. Ecol Lett. 20:275–92. 28090753 10.1111/ele.12732 PMC7163542

[bib93] Petruk G , Puthia M, Petrlova J, Samsudin F, Strömdahl A-C, Cerps S, Uller L, Kjellström S, Bond PJ, Schmidtchen AA. 2020. SARS-CoV-2 spike protein binds to bacterial lipopolysaccharide and boosts proinflammatory activity. J Mol Cell Biol. 12:916–32. 33295606 10.1093/jmcb/mjaa067 PMC7799037

[bib94] Picotti P , Aebersold R. 2012. Selected reaction monitoring-based proteomics: workflows, potential, pitfalls and future directions. Nat Methods. 9:555–66. 22669653 10.1038/nmeth.2015

[bib95] Plowright RK , Sokolow SH, Gorman ME. 2008. Causal inference in disease ecology: investigating ecological drivers of disease emergence. Front Ecol Environ. 6:420–9.,.

[bib96] Qu X , Zhu Z, Zhou X, Wu X, Liu X, Sun X, Zhang J, Du G, Xue R, Zhang Q et al. 2025. KRT9 is required for GBP5 suppression of human respiratory syncytial virus. J Virol. 99:e0202924. 39835811 10.1128/jvi.02029-24 PMC11852966

[bib97] Ramlall V , Thangaraj PM, Meydan C, Foox J, Butler D, Kim J, May B, De Freitas JK, Glicksberg BS, Mason CE et al. 2020. Immune complement and coagulation dysfunction in adverse outcomes of SARS-CoV-2 infection. Nat Med. 26:1609–15. 32747830 10.1038/s41591-020-1021-2 PMC7809634

[bib98] Raudvere U , Kolberg L, Kuzmin I, Arak T, Adler P, Peterson H, Vilo J. 2019. g:Profiler: a web server for functional enrichment analysis and conversions of gene lists (2019 update). Nucleic Acids Res. 47:W191–98. 31066453 10.1093/nar/gkz369 PMC6602461

[bib99] Robin X , Turck N, Hainard A, Tiberti N, Lisacek F, Sanchez J-C, Müller M. 2011. pROC: an open-source package for R and S+ to analyze and compare ROC curves. BMC Bioinf. 12:77. 10.1186/1471-2105-12-77 PMC3068975 21414208

[bib100] Russell AL , Cox MP, Brown VA, McCracken GF. 2011. Population growth of Mexican free-tailed bats (*Tadarida brasiliensis mexicana*) predates human agricultural activity. BMC Evol Biol. 11:88. 21457563 10.1186/1471-2148-11-88 PMC3080819

[bib101] Russell AL , Medellín RA, McCracken GF. 2005. Genetic variation and migration in the Mexican free-tailed bat (*Tadarida brasiliensis mexicana*). Mol Ecol. 14:2207–22. 15910338 10.1111/j.1365-294X.2005.02552.x

[bib102] Salazar F , Bignell E, Brown GD, Cook PC, Warris A. 2022. Pathogenesis of respiratory viral and fungal coinfections. Clin Microbiol Rev. 35:e0009421. 34788127 10.1128/CMR.00094-21 PMC8597983

[bib103] Schmid DW , Meyer M, Wilhelm K, Tilley T, Link-Hessing T, Fleischer R, Badu EK, Nkrumah EE, Oppong SK, Schwensow N et al. 2023. MHC class II genes mediate susceptibility and resistance to coronavirus infections in bats. Mol Ecol. 32:3989–4002. 37203872 10.1111/mec.16983

[bib104] Schwartz A . 1955. The status of the species of the *Brasiliensis* group of the genus *Tadarida*. J Mammal. 36:106–9.

[bib105] Sikes RS , Gannon WL. 2011. Guidelines of the American Society of Mammalogists for the use of wild mammals in research. J Mammal. 92:235–53. 10.1093/jmammal/gyw078 PMC5909806 29692469

[bib106] Sim M , Lee J, Lee D, Kwon D, Kim J. 2020. TAMA: improved metagenomic sequence classification through meta-analysis. BMC Bioinf. 21:185. 10.1186/s12859-020-3533-7 PMC7218625 32397982

[bib107] Simão FA , Waterhouse RM, Ioannidis P, Kriventseva EV, Zdobnov EM. 2015. BUSCO: assessing genome assembly and annotation completeness with single-copy orthologs. Bioinformatics. 31:3210–2. 26059717 10.1093/bioinformatics/btv351

[bib108] Sit THC , Brackman CJ, Ip SM, Tam KWS, Law PYT, To EMW, Yu VYT, Sims LD, Tsang DNC, Chu DKW et al. 2020. Infection of dogs with SARS-CoV-2. Nature. 586:776–8. 32408337 10.1038/s41586-020-2334-5 PMC7606701

[bib109] Song J , Choi J, Park L. et al. 2024. Optimization of automated suspension trapping digestion in bottom-up proteomics via mass spectrometry. Mass Spectrom Lett. 15:62–8.

[bib110] Stepanian PM , Wainwright CE. 2018. Ongoing changes in migration phenology and winter residency at Bracken Bat Cave. Glob Chang Biol. 24:3266–75. 29442413 10.1111/gcb.14051

[bib111] Sweeny AR , Albery GF, Becker DJ, Eskew EA, Carlson CJ. 2021. Synzootics. J Anim Ecol. 90:2744–54. 34546566 10.1111/1365-2656.13595

[bib112] Teeling EC , Vernes SC, Dávalos LM, Ray DA, Gilbert MTP, Myers E, Bat1K Consortium. 2018. Bat biology, genomes, and the Bat1K project: to generate chromosome-level genomes for all living Bat species. Annu Rev Anim Biosci. 6:23–46. 29166127 10.1146/annurev-animal-022516-022811

[bib113] Uhlén M , Karlsson MJ, Hober A, Svensson A-S, Scheffel J, Kotol D, Zhong W, Tebani A, Strandberg L, Edfors F et al. 2019. The human secretome. Sci Signal. 12:eaaz0274. 31772123 10.1126/scisignal.aaz0274

[bib114] Vicente-Santos A , Lock LR, Allira M, Dyer KE, Dunsmore A, Tu W, Volokhov DV, Herrera C, Lei G-S, Relich RF et al. 2023. Serum proteomics reveals a tolerant immune phenotype across multiple pathogen taxa in wild vampire bats. Front Immunol. 14:1281732. 38193073 10.3389/fimmu.2023.1281732 PMC10773587

[bib115] Villa BR , Cockrum EL. 1962. Migration in the guano bat *Tadarida brasiliensis mexicana* (Saussure). J Mammal. 43:43–64.

[bib116] Webb-Robertson B-JM , Wiberg HK, Matzke MM, Brown JN, Wang J, McDermott JE, Smith RD, Rodland KD, Metz TO, Pounds JG et al. 2015. Review, evaluation, and discussion of the challenges of missing value imputation for mass spectrometry-based label-free global proteomics. J Proteome Res. 14:1993–2001. 25855118 10.1021/pr501138h PMC4776766

[bib117] Webster CF , Smotherman M, Pippel M, Brown T, Winkler S, Pieri M, Mai M, Myers EW, Teeling EC, Vernes SC et al. 2024. The genome sequence of *Tadarida brasiliensis* I. Geoffroy Saint-Hilaire, 1824 [Molossidae; Tadarida]. Wellcome Open Res. 9:98. 38800517 10.12688/wellcomeopenres.20603.1 PMC11128047

[bib118] Wiederholt R , López-Hoffman L, Cline J. RA Medellín, P Cryan, A Russell, G McCracken, J Diffendorfer, D Semmens 2013. Moving across the border: modeling migratory bat populations.Ecosphere. 4:1–6.

[bib119] Woo PCY , Lau SKP, Lam CSF, Lau CCY, Tsang AKL, Lau JHN, Bai R, Teng JLL, Tsang CCC, Wang M et al. 2012. Discovery of seven novel mammalian and avian coronaviruses in the genus deltacoronavirus supports bat coronaviruses as the gene source of *Alphacoronavirus* and *Betacoronavirus* and avian coronaviruses as the gene source of *Gammacoronavirus* and *Deltacoronavirus*. J Virol. 86:3995–4008. 22278237 10.1128/JVI.06540-11 PMC3302495

[bib120] Woon AP , Boyd V, Todd S, Smith I, Klein R, Woodhouse IB, Riddell S, Crameri G, Bingham J, Wang L-F et al. 2020. Acute experimental infection of bats and ferrets with Hendra virus: insights into the early host response of the reservoir host and susceptible model species. PLoS Pathog. 16:e1008412. 32226041 10.1371/journal.ppat.1008412 PMC7145190

[bib121] Xiu L , Binder RA, Alarja NA, Kochek K, Coleman KK, Than ST, Bailey ES, Bui VN, Toh T-H, Erdman DD et al. 2020. A RT-PCR assay for the detection of coronaviruses from four genera. J Clin Virol. 128:104391. 32403008 10.1016/j.jcv.2020.104391 PMC7192118

[bib122] Zhang Q , Zhang H, Gao J, Huang K, Yang Y, Hui X, He X, Li C, Gong W, Zhang Y et al. 2020. A serological survey of SARS-CoV-2 in cat in Wuhan. Emerg Microbes Infect. 9:2013–9. 32867625 10.1080/22221751.2020.1817796 PMC7534315

[bib123] Zhou P , Yang X-L, Wang X-G, Hu B, Zhang L, Zhang W, Si H-R, Zhu Y, Li B, Huang C-L et al. 2020. A pneumonia outbreak associated with a new coronavirus of probable bat origin. Nature. 579:270–3. 32015507 10.1038/s41586-020-2012-7 PMC7095418

