# Correction to: Proteomic Signatures of SARS-CoV-2 Susceptibility in Mexican Free-tailed Bats and Their Application to Viral Surveillance

**DOI:** 10.1093/icb/icaf156

**Published:** 2025-11-20

This is a correction to Daniel J Becker, Amanda Vicente-Santos, Ariadna E Morales, Kristin E Dyer, Beckett L Olbrys, Lauren R Lock, Michael S Smotherman, Sonja C Vernes, Michael Hiller, Amanda M Adams, Brett S Phinney, Winifred F Frick, Jeffrey S Hall, Proteomic Signatures of SARS-CoV-2 Susceptibility in Mexican Free-tailed Bats and Their Application to Viral Surveillance, *Integrative and Comparative Biology*, 2025, icaf148, https://doi.org/10.1093/icb/icaf148.

Following article publication, the authors asked both to remove the specific sampling locality throughout the text and to modify Fig. 3 for conservation reasons. The Editor agrees with the corresponding author’s assertion that these changes do not affect the results or conclusions of the paper.

