# Supplementary material for: Proteomic Signatures of SARS-CoV-2 Susceptibility in Mexican Free-tailed Bats and Their Application to Viral Surveillance
Source: Integr Comp Biol. 2025 Aug 15;65(6):1884–900. doi: 10.1093/icb/icaf148 (PMC12690474; doi:10.1093/icb/icaf148)

Proteomic signatures of SARS-CoV-2 susceptibility in Mexican free-tailed bats and their application to viral surveillance: Supplemental Material

Daniel J. Becker, Amanda Vicente-Santos, Ariadna E. Morales, Kristin E. Dyer, Beckett L. Olbrys, Lauren R. Lock, Michael S. Smotherman, Sonja C. Vernes, Michael Hiller, Amanda M. Adams, Brett S. Phinney, Winifred F. Frick, Jeffrey S. Hall

Additional DIA-NN data processing details
Figure S1: Proteomic comparison between common vampire bats and Mexican free-tailed bats
Figure S2: Dynamic range of the Mexican free-tailed bat plasma proteome
Figure S3: Proteomic comparison between wild and captive Mexican free-tailed bats
Figure S4: Proteome composition between wild and captive Mexican free-tailed bats
Figure S5: Proteome composition and LFC between susceptible and non-susceptible bats
Figure S6: Enrichment analysis of candidate protein biomarkers of SARS-CoV-2 susceptibility
Table S1: CoV positivity data in wild Mexican free-tailed bats (March 2022), stratified by swab
Figure S7: Comparison of Mexican free-tailed bat plasma and human blood protein ranks

Additional DIA-NN data processing details

The raw Bruker .d files were used directly in DIA-NN (v2.1.0; Demichev et al. 2020) rather than preconverting to mzML or other formats. The *Tadarida brasiliensis* protein fasta was made to mimic the UniProt header format to limit issues related to how protein grouping was performed. The following parameters were used under "Precursor ion generation": fasta digest for library search; deep learning-based spectra; RTs and Ims prediction were selected; Trypsin/P was selected for the protease, allowing one mis-cleavage; maximum number of variable modifications was set to 1; N-term M excision and C carbamidomethylation were selected; peptide length range was set to 7 to 30; precursor charge range was set to 1 to 4;, precursor m/z range was set to 300 to 1800; and fragment ion m/z range was set to to 200 to 1800. These are all default settings, suggested as appropriate in the DIA-NN documentation. The precursor FDR was set to 1 %. The algorithm settings were set to mass accuracy of 10.0 ppm and MS1 accuracy of 15.0 ppm, according to documentation recommendations for timsTOF data. The scan window was set to 0, and unrelated runs were selected to allow DIA-NN to determine the scan window scheme in the DIA method. The options for MBR and protein inference were checked. The scoring used was peptidoforms, proteotypicity set to genes, machine learning to NNs (cross-validated), quantification strategy set to QuantUMS (high precision) (Kistner et al. 2023), cross-run normalization to RT-dependent, library generation to IDs, RT and IM profiling, and speed and RAM usage to optimal result. All of these are default settings, except for Mass and MS1 accuracy, Scan window, and unrelated runs. For the final data output, the report.unique_genes_matrix.tsv was used, where pg is protein groups and gg is gene groups, which are defined as gene names corresponding to the proteins in the group. Since different proteins may come from the same gene, the pg (protein group) and gg (gene group) matrices are different. Using the unique genes matrix is the same as the gene group matrix but quantified with proteotypic peptides only. All of the matrix has the 1 % global FDR filter specified in place.

Kistner F, Grossmann JL, Sinn LR, Demichev V. 2023. QuantUMS: uncertainty minimisation enables confident quantification in proteomics. bioRxiv.

Figure S1. Shared and unique proteins between our previously published common vampire bat (*Desmodus rotundus*) serum proteome (Becker et al. 2022) and the plasma proteome of Mexican free-tailed bats (*Tadarida brasiliensis*) here. Captive and wild bat proteomes are pooled.

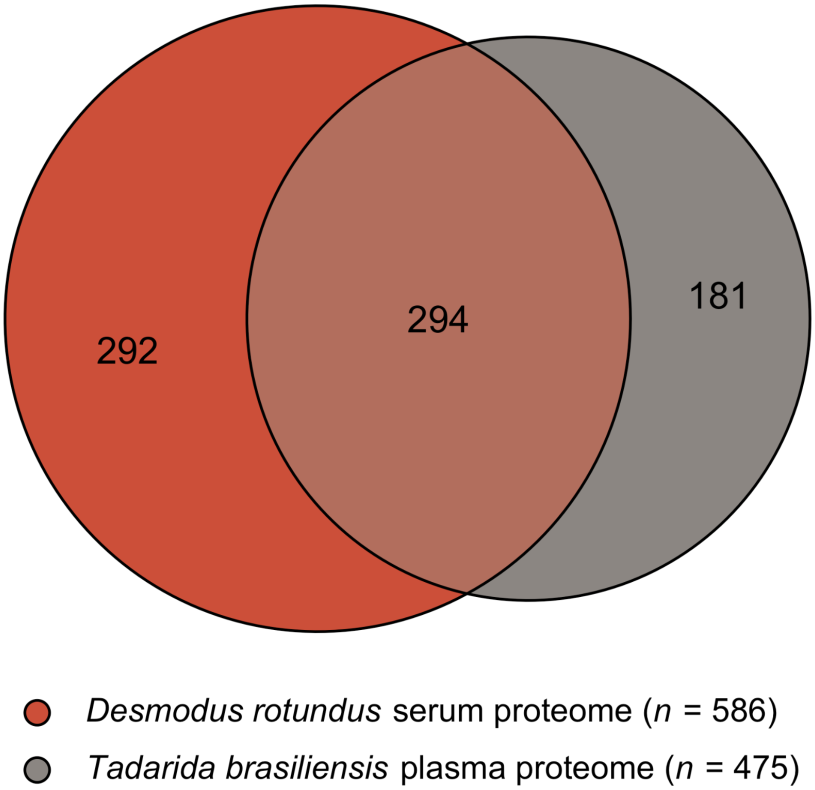

Figure S2. Dynamic range of the Mexican free-tailed bat plasma proteome and top 20 most abundant proteins, combining both captive and wild bat data. Proteins that were previously identified in the common vampire bat serum proteome (Becker et al. 2022; red) are distinguished from those specific to only the Mexican free-tailed bat (grey).

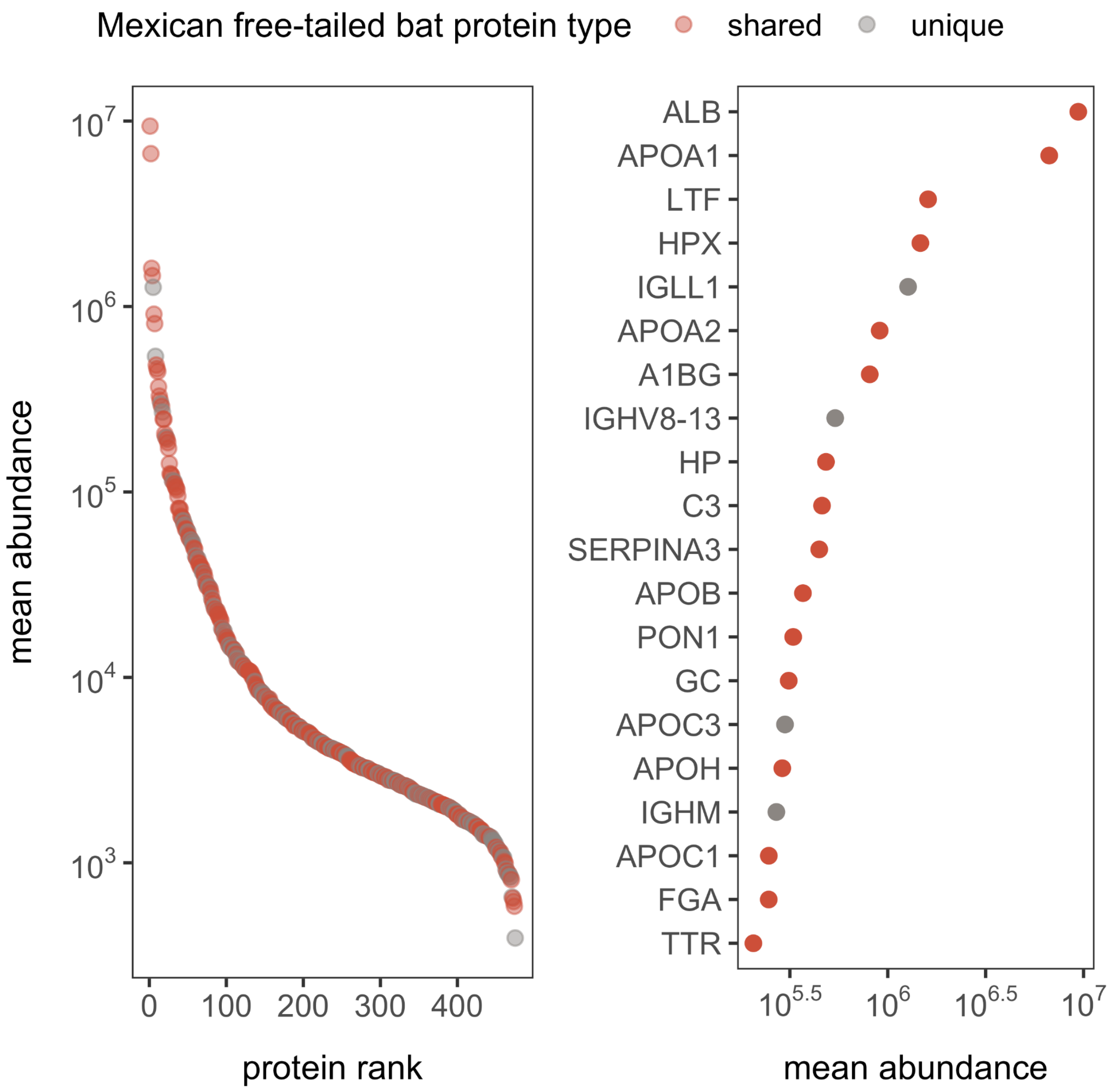

Figure S3. Shared and unique plasma proteins between captive (brown) and wild (grey) Mexican free-tailed bat populations from Texas.

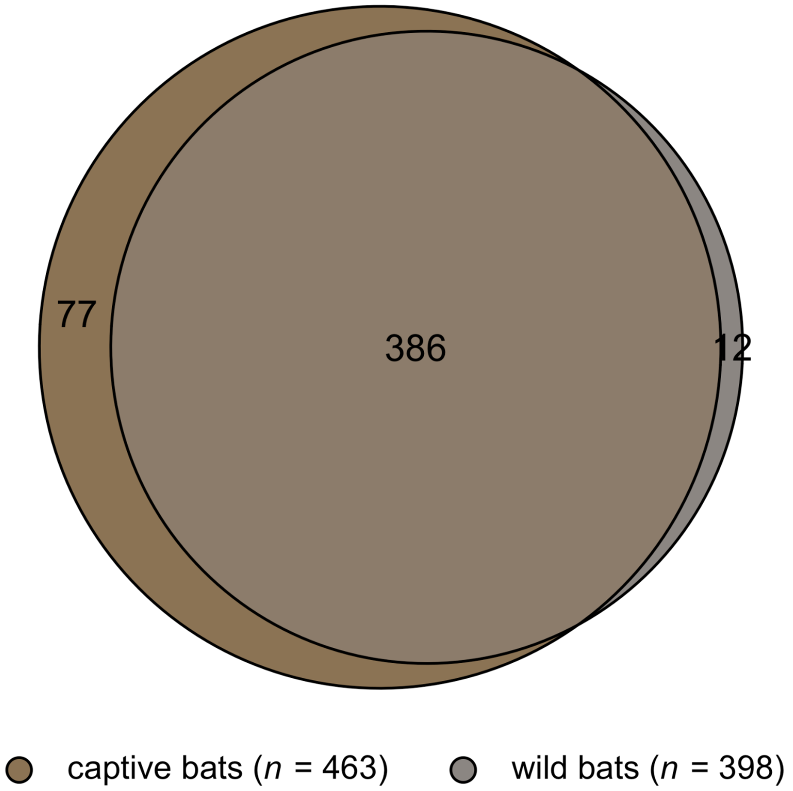

Figure S4. Principal components analysis (PCA) of the abundance of the 475 plasma proteins found in captive (brown) and wild (grey) Mexican free-tailed bats. Points represent individual bats, with ellipses showing 95% confidence intervals; both are colored by bat population.

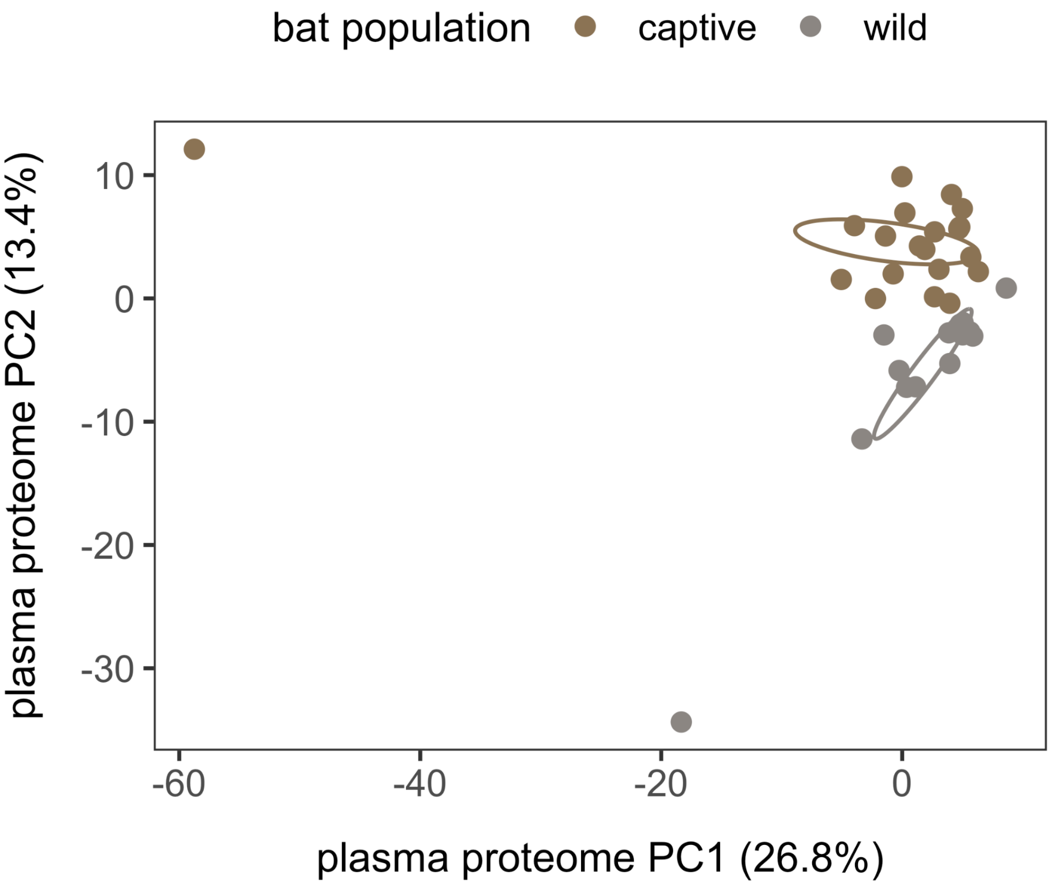

Figure S5. Top: Principal components analysis (PCA) of the abundance of the 446 proteins found in captive Mexican free-tailed bats challenged with SARS-CoV-2. Points represent individuals, with ellipses showing 95% confidence intervals; both are colored by post-challenge infection. Bottom: Comparison of mean protein abundance for non-susceptible and susceptible bats. The dashed line shows the 1:1 reference, and points are colored by absolute $\log_2$-fold change (LFC).

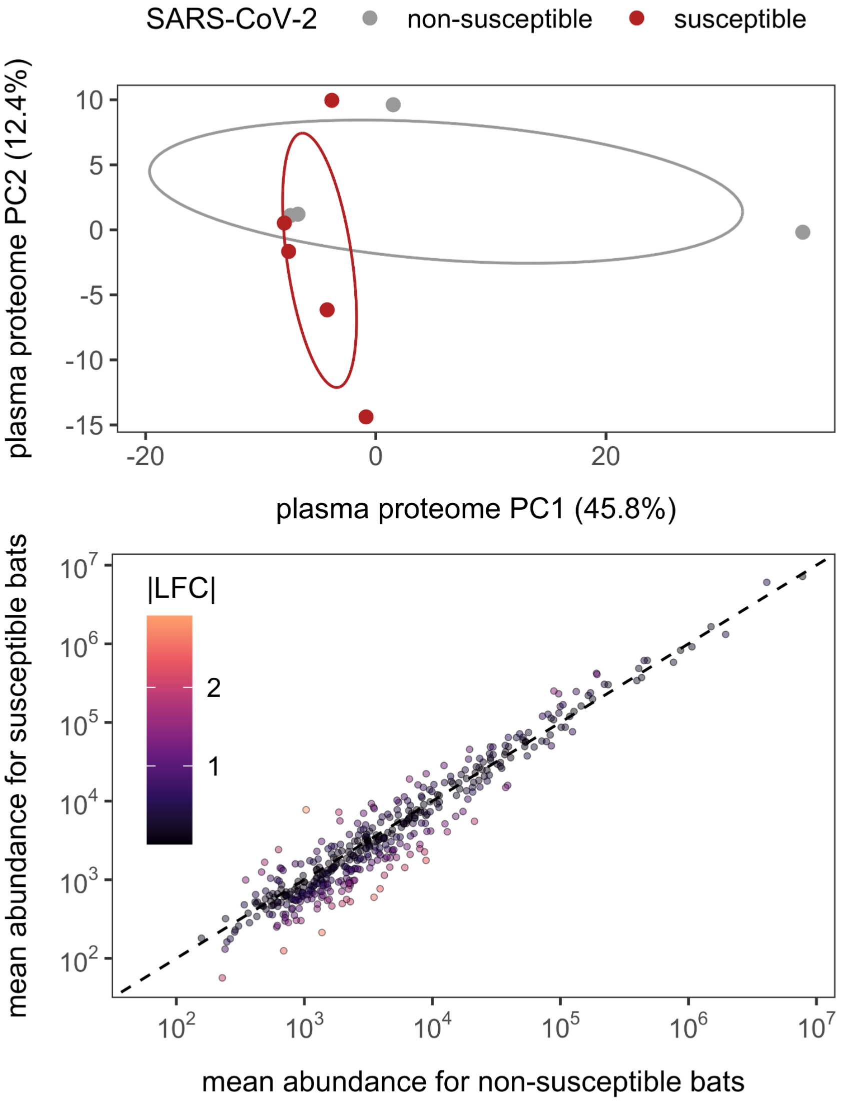

Figure S6. Enrichment analyses of the 27 candidate biomarkers of SARS-CoV-2 susceptibility. Biological processes with significant enrichment in susceptible bats after SCS correction are displayed, with up- and down-regulated processes shown in red and blue, respectively. Points are scaled by term size (dashed line shows $p = 0.05$).

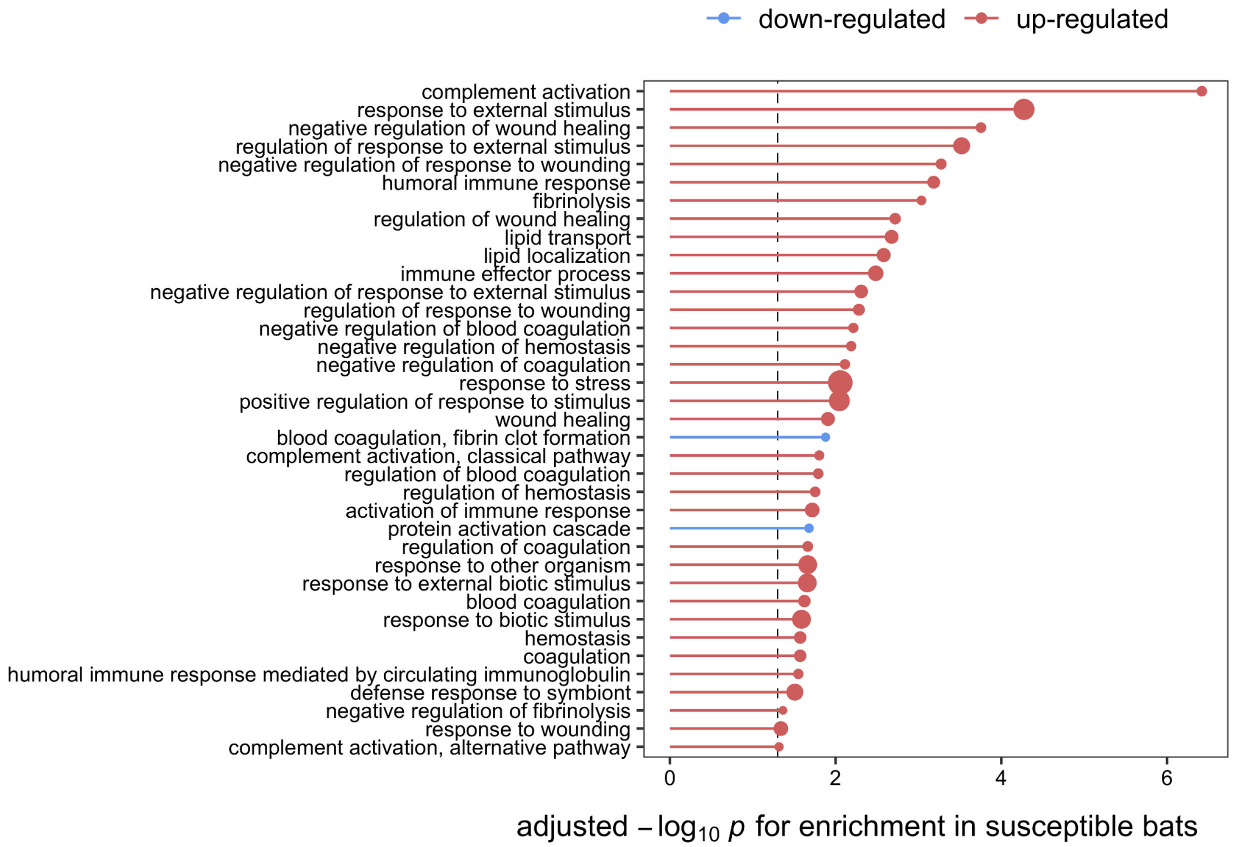

Table S1. CoV positivity data in wild Mexican free-tailed bats (March 2022), stratified by swab.

ID	Oral swab	Rectal swab	GenBank
TX1	Negative	Negative	
TX2	Negative	β-CoV	PV540131
TX3	Negative	Negative	
TX4	Negative	Negative	
TX5	Negative	Negative	
TX6	Negative	Negative	
TX7	Negative	Negative	
TX8	Negative	Negative	
TX9	Negative	Negative	
TX10	Negative	Negative	
TX11	Negative	Negative	
TX12	Negative	Negative	
TX13	Negative	Negative	
TX14	Negative	Negative	
TX15	Negative	Negative	
TX16	Negative	Negative	
TX17	α-CoV	Negative	PV540130
TX18	α-CoV	Negative	PV540129
TX19	β-CoV	Negative	PV540128
TX20	Negative	Negative	

Figure S7. Comparison of Mexican free-tailed bat plasma and human blood protein ranks. Human data are derived from the Human Protein Atlas. Red indicates the rank of KRT1, and dashed lines show the 40th percentile for each set of protein ranks. 435 of the 475 proteins in the Mexican free-tailed bat plasma proteome are detectable in humans.

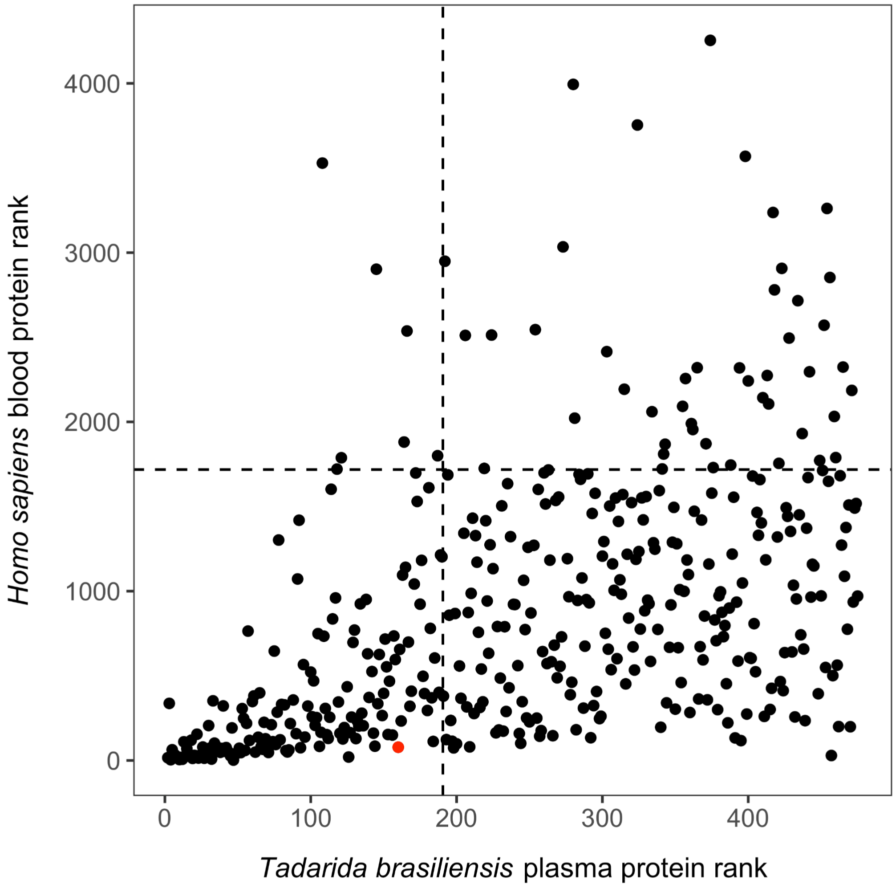

Supplement: icaf148_Supplemental_File [file icaf148_supplemental_file.pdf]
